# Adjuvant Systemic Therapy after Chemoradiation and Brachytherapy for Locally Advanced Cervical Cancer: A Systematic Review and Meta-Analysis

**DOI:** 10.3390/cancers13081880

**Published:** 2021-04-14

**Authors:** Nanda Horeweg, Prachi Mittal, Patrycja L. Gradowska, Ingrid Boere, Supriya Chopra, Remi A. Nout

**Affiliations:** 1 Department of Radiation Oncology, Leiden University Medical Center, 2333ZA Leiden, The Netherlands; 2 Department of Radiation Oncology, Tata Memorial Hospital, Tata Memorial Centre, Parel, Homi Bhabha National Institute, Mumbai 400094, India; mittalprachi@gmail.com; 3 Department of Hematology—HOVON Data Center, Erasmus MC Cancer Institute, P.O. box 2040, 3000CA Rotterdam, The Netherlands; p.gradowska@erasmusmc.nl; 4 Department of Medical Oncology, Erasmus Medical Center Cancer Institute, P.O. box 2040, 3000CA Rotterdam, The Netherlands; i.boere@erasmusmc.nl; 5 Department of Radiation Oncology, Advanced Centre for Treatment, Research and Education in Cancer, Tata Memorial Centre, Parel, Homi Bhabha National Institute, Mumbai 400094, India; schopra@actrec.gov.in; 6 Department of Radiotherapy, Erasmus MC Cancer Institute, University Medical Center, P.O. box 2040, 3000CA Rotterdam, The Netherlands; r.nout@erasmusmc.nl

**Keywords:** meta-analysis, cervical cancer, adjuvant therapy, chemotherapy, immunotherapy, overall survival

## Abstract

**Simple Summary:**

The standard of care for locally advanced cervical cancer is chemoradiation and brachytherapy. The addition of adjuvant systemic treatment may improve overall survival. A systematic review and meta-analysis were conducted to summarize evidence on survival outcomes, treatment completion and toxicity. Thirty-five articles reporting on 29 different studies were selected from a total of 612 articles published on this topic since 2000. Twelve studies on two different chemotherapy combinations (platinum–pyrimidine antagonist and platinum–taxane) were included for meta-analysis. Both these adjuvant chemotherapy combinations did not yield a survival benefit but did lead to more severe side-effects than chemoradiation only. Therefore, these adjuvant treatment strategies cannot be recommended for unselected patients with locally advanced cervical cancer. Most of the studies on other chemotherapeutic agents did not seem to provide a good balance between efficacy and toxicity either. The evidence on adjuvant immunotherapy for locally advanced cervical cancer is still immature.

**Abstract:**

Background: Standard of care for locally advanced cervical cancer is chemoradiation and brachytherapy. The addition of adjuvant systemic treatment may improve overall survival. A systematic review and meta-analysis was conducted to summarize evidence on survival outcomes, treatment completion and toxicity. Methods: PubMed, EMBASE and Web of Science were systematically searched for relevant prospective and retrospective studies. Two authors independently selected studies, extracted data and assessed study quality. Pooled hazard ratios for survival endpoints were estimated using random effect models. Weighted averages of treatment completion and toxicity rates were calculated and compared by the Fisher exact test. Results: The search returned 612 articles; 35 articles reporting on 29 different studies on adjuvant chemotherapy or immunotherapy were selected for systematic review. Twelve studies on an adjuvant platinum–pyrimidine antagonist or platinum–taxane were included for meta-analysis. The pooled hazard ratios for overall survival were 0.76 (99%CI: 0.43–1.34, *p* = 0.22) and 0.47 (99%CI: 0.12–1.86, *p* = 0.16) for the addition of, respectively, a platinum–pyrimidine antagonist or platinum–taxane to chemoradiation and brachytherapy. Completion rates were 82% (95%CI: 76–87%) for platinum–pyrimidine antagonist and 74% (95%CI: 63–85%) for platinum–taxane. Severe acute hematological and gastro-intestinal toxicities were significantly increased by adding adjuvant chemotherapy to chemoradiation and brachytherapy. Conclusions: The addition of adjuvant platinum–pyrimidine antagonist or platinum–taxane after chemoradiation and brachytherapy does not significantly improve overall survival, while acute toxicity is significantly increased. These adjuvant treatment strategies can therefore not be recommended for unselected patients with locally advanced cervical cancer.

## 1. Introduction

Cervical cancer is the second most common cancer in women across the world [1]. The standard of care for locally advanced cervical cancer has been platinum-based chemoradiation with brachytherapy since the National Cancer Institute alert in 1999 [2]. A meta-analysis of 13 randomized controlled trials showed a 6% improvement in 5-year overall survival by adding concurrent chemotherapy to radiation [3]. In recent years, further improvement of overall survival was reported with image-based brachytherapy and radiation dose escalation, while reducing toxicity [4,5,6,7,8]. These treatment advances changed patterns of failure. Distant metastases are now the most common type of failure, occurring in 24–30% at 5 years after chemoradiation and brachytherapy [9,10]. Distant metastases occur due to the incomplete eradication of the primary tumor or involved lymph nodes or due to undetected micro-metastasis outside the field of treatment [9]. Adjuvant systemic therapy after chemoradiation and brachytherapy has the potential to reduce the risk of distant metastasis and improve overall survival.

A sub-analysis of the aforementioned meta-analysis showed that concurrent chemoradiation with adjuvant chemotherapy yielded a 19% 5-year overall survival benefit compared to radiotherapy alone [3]. Since chemoradiation is nowadays the standard of care, an important question is whether and to what extent overall survival is improved by chemoradiation followed by adjuvant systemic therapy compared to chemoradiation. A randomized controlled trial by Duenas-Gonzales et al. on radiotherapy with concurrent cisplatin vs. radiotherapy with concurrent cisplatin–gemcitabine followed by adjuvant cisplatin–gemcitabine showed a significant improvement in overall survival (hazard ratio (HR) 0.68, 95% confidence interval (CI): 0.49–0.95, *p* = 0.02). However, this trial could not give an answer as the treatment groups did not receive the same concurrent chemotherapy [11]. A 2014 Cochrane review was also not able to answer this question because only two randomized controlled trials on chemoradiation followed by adjuvant chemotherapy vs. chemoradiation were found and not pooled [12].

An overview of all clinical studies on adjuvant systemic therapy after chemoradiation and brachytherapy is therefore needed to summarize the impact, if any, on disease-related outcomes and to provide direction for the design of future trials. We performed a systematic review and meta-analysis to provide this overview and pooled estimates of the efficacy and toxicity of adjuvant systemic therapy after chemoradiation and brachytherapy for locally advanced cervical cancer.

## 2. Materials and Methods

### 2.1. Article Characteristics

The design of the systematic search is presented in Table 1. Articles on randomized and non-randomized prospective and retrospective studies were eligible if chemoradiation with brachytherapy followed by adjuvant systemic therapy was investigated or compared to standard chemoradiation with brachytherapy. The following article types were not eligible: conference abstracts, case-reports, review articles, meta-analyses, editorials, letters to editor and guidelines.

Articles published before the year 2000 were excluded because concurrent chemoradiation was not established as the standard of treatment before the National Cancer Institute alert [2].

### 2.2. Study Population Characteristics

Patients with a diagnosis of Fédération Internationale de Gynécologie et d’Obstétrique (FIGO) stage IB–IVA cervical cancer of the squamous cell carcinoma, adenocarcinoma or adenosquamous carcinoma histological type were included. 

Studies including patients with proven or suspected metastasis to para-aortic lymph nodes were eligible. Studies on patients who were treated with primary surgery and adjuvant chemoradiation and systemic chemotherapy were not included. Neither did we include studies of patients with distant metastasis or with persistent or recurrent cervical cancer after failure of previous treatment(s).

### 2.3. Treatment Characteristics

Studies were eligible if external beam radiotherapy was delivered to the whole pelvis with or without integrated or sequential boost(s). Extended field external beam radiotherapy for involved or suspected para-aortic lymph nodes or as prophylactic treatment was allowed. Concurrent chemotherapy was preferably platinum-based, but other concurrent agents were accepted. During or after chemoradiation, patients had to undergo intracavitary and/or interstitial brachytherapy. Adjuvant systemic therapy had to consist of at least one administration after chemoradiotherapy and brachytherapy of any systemically active agent, e.g., chemotherapy, monoclonal antibodies, immunomodulators, and the intent of treatment had to be curative.

### 2.4. Outcomes Measures

Studies had to report on ≥1 of the following outcomes: distant metastasis-free survival; recurrence or disease-free survival; overall survival; treatment completion; and toxicity. These outcomes had to be reported separately for the patients undergoing chemoradiation and those undergoing chemoradiation followed by adjuvant systemic therapy as applicable.

### 2.5. Literature Searches

Search strings for PubMed, Web of Science and EMBASE were devised by N.H. with assistance from a trained librarian (provided in Supplementary Materials I), to identify relevant studies published until 5 September 2020. The following terms, and possible variations thereof, were matched to appropriate medical subject headings: “cervical cancer”; “chemoradiation” and “adjuvant therapy”. Searches were restricted to publications in or after the year 2000. Study authors were contacted if full texts were not available. Grey literature sources, such as clinicaltrials.gov and Google Scholar, were searched for ongoing and unpublished trials.

### 2.6. Selection of Studies

All articles were imported in EndNote X9 and deduplicated before study selection. Two reviewers (N.H. and P.M.) independently read the titles and abstracts of all articles to identify relevant studies for full text review. Hand searches of reference lists of the articles selected for full text review were performed to identify additional relevant articles. At every stage of the selection process, the independent results of the two review authors were compared and any differences were solved in consensus meetings or by the decision of a third reviewer (S.C.). All selected studies were included in the systematic review. The inclusion in the meta-analysis was possible if there were two or more articles on chemoradiation and brachytherapy vs. chemoradiation and brachytherapy followed by a systemic agent (combination).

If ≥1 article described the same study, the most recent and complete article was used for analysis. However, if efficacy and toxicity outcomes were reported in two separate articles, both were included.

### 2.7. Risk of Bias Assessment

A pre-specified risk of bias assessment (provided in the Supplementary Materials) was based on the Cochrane Handbook for Systematic Reviews version 5.1.0 [13], and the “Meta-analysis Of Observational Studies in Epidemiology” consensus on reporting of observational studies [14]. Both review authors (N.H. and P.M.) were trained and a pilot study with a test article was conducted. For each study, the two reviewers independently rated all risk of bias aspects. Studies with discrepancies were listed and discussed, and in case of remaining disagreement, the third reviewer was consulted. A study’s overall risk of bias was classified as: (1) low if the risk of bias was low for all domains; (2) some concerns if there were unclarities or some concerns of risk of bias in one domain; (3) high if there was a high risk of bias in ≥1 domain. The overall risk of bias will be reported along with the outcomes of the included studies.

### 2.8. Data Extraction

The data extraction protocol is provided in the Supplementary Materials I. The following pre-specified information was extracted: publication details, study design and population, treatment and summary measures of outcomes. The latter consisted of follow-up time, treatment completion rates, survival outcomes (2- and 3-year estimates, hazard ratios with 95% confidence intervals, acute (occurring <3 months) and late (occurring or persisting beyond 3 months) severe toxicity (grade 3–5). If survival outcomes were not directly reported, the estimates were deducted from survival graphs or reported crude numbers. Extracted data were compared, variables with discrepancies were listed and discussed by the two reviewers and with the third reviewer in case of disagreement.

### 2.9. Statistical Methods

The primary outcome is overall survival. Secondary outcomes are distant metastasis-free survival and recurrence-free survival, treatment completion rate and the rates of severe acute and late toxicities. 

Overall survival time is defined as the time from the date of inclusion/randomization to date of death, or date of last follow-up in alive patients. Recurrence-free survival time is defined as the time from the date of inclusion/randomization to the date of first recurrence (regardless of localization), or the date of last follow-up in patients without recurrence. Metastasis-free survival time is defined as the time from date of inclusion/randomization to date of first distant metastasis (recurrence beyond pelvis or para-aortic lymph node chain), or the date of last follow-up in patients without recurrence. Severe toxicities were defined as grade ≥3 according to the Common Terminology Criteria for Adverse Events or the Radiation Therapy Oncology Group/European Organisation for Research and Treatment of Cancer classification, as reported in the original articles. 

Total radiotherapy doses were calculated as doses equivalent to 2 Gy fractions (EQD2) using an α/β = 10. Pooled estimates of radiotherapy dose, treatment completion and toxicity rates were calculated as weighted averages with 95% CIs and compared with the Fisher exact test. 

For the survival endpoints, if included studies reported HRs and 95% CIs, the natural logarithm of HR and its variance were calculated directly (Supplementary Materials I). If not, these were imputed using other data provided in the article according to the methodology of Tierney et al. (Supplementary Materials I) [15]. Pooled estimates of the hazard ratios for overall survival, distant metastasis-free survival and recurrence-free survival were calculated using random effects models (DerSimonian–Laird method) wherein each study is weighed according to their sample size. Separate models were built for each adjuvant systemic treatment for which ≥2 studies reported survival outcomes. Random-effects models were chosen a priori because of the anticipated clinical and methodological heterogeneity between the studies. The level of statistical significance was pre-defined as *p* < 0.01 to correct for multiple testing. Heterogeneity, in effect size among studies, was assessed by the I^2^ and the Q-test. Significant statistical heterogeneity between studies was defined as an I^2^ > 50% with the Q-test *p* < 0.05. Heterogeneity due to of pooling studies with different designs was addressed by pre-specified subgroup analyses (randomized controlled trials vs. non-randomized controlled trials). Pre-specified sensitivity analyses consisted of re-estimating all pooled estimates according to the leave-one-out method using random effect models, to evaluate whether the results could have been affected markedly by a single study. Publication bias was assessed using funnel plots in the meta-analysis for the primary endpoint. Descriptive analyses were used if the data were limited.

Analyses were performed in Microsoft Excel and R version 3.6.1 (http://www.r-project.org/ (accessed on 8 April 2021)). R packages used in this study are reported in the Supplementary Materials.

This study was registered at PROSPERO under registration number CRD42020211194 and conducted according to the Preferred Reporting Items for Systematic Review and Meta-Analysis guidelines [16].

## 3. Results

### 3.1. Systematic Searches

**Figure 1:**
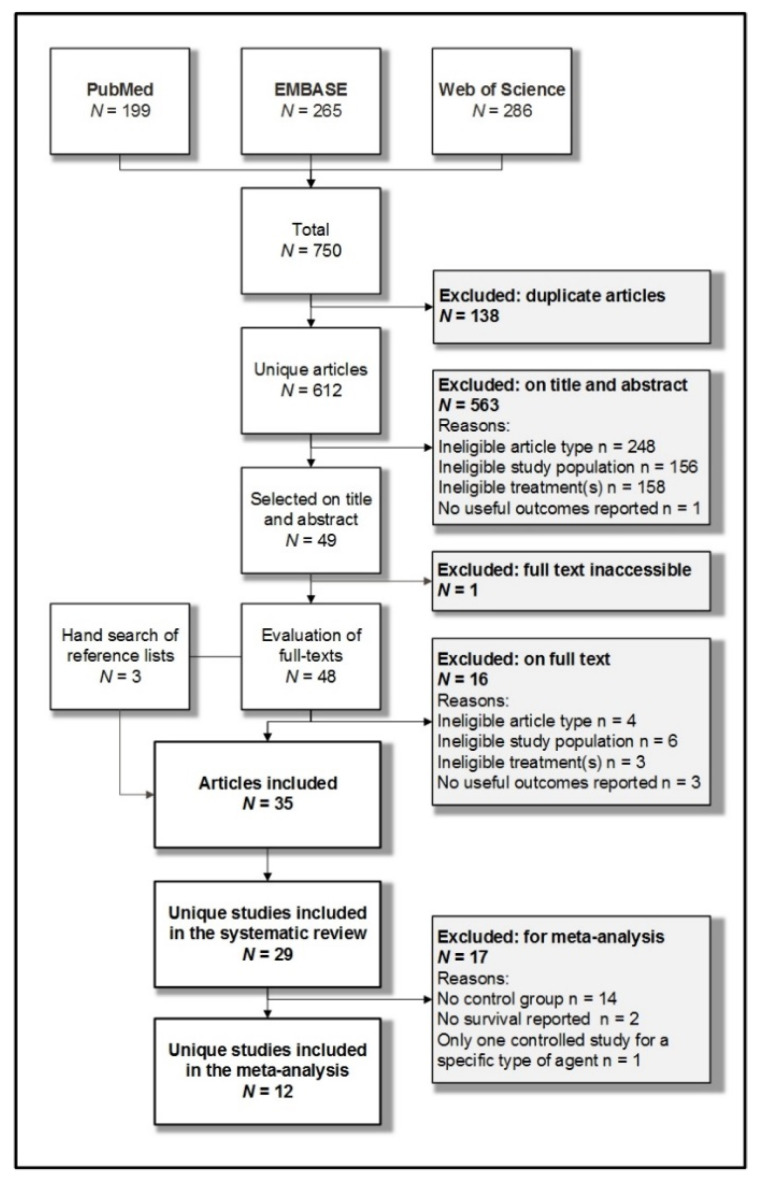
Systematic search and article selection process.

Systematic searches yielded 612 unique articles (Figure 1). Forty-nine were selected for full-text review, of which 32 were eligible for inclusion. Hand searches of reference lists yielded another three eligible articles. These 35 articles reported on 29 different studies and were included in the systematic review. Twelve of 29 reported studies were also included in the meta-analysis. Reasons for the exclusion of the remaining 17 studies are listed in Figure 1.

### 3.2. Characteristics Included Studies

Table 2 shows the main characteristics of the 29 studies included in the systematic review. Fifteen of the 29 included studies compared chemoradiation followed by adjuvant systemic therapy to chemoradiation alone using various designs. The remaining 14 studies that reported on chemoradiation followed by adjuvant systemic therapy did not have a control group treated with chemoradiation.

Tables S1 and S2 in supplemental data II describe the radiotherapy techniques used in the included studies. Generally, external beam radiotherapy was conventionally planned using computed tomography and delivered by parallel opposing or box techniques. The use of extended field external beam radiotherapy for positive para-aortic lymph nodes was reported in 45% of the studies and prophylactic extended field was reported in 17% of the studies. Brachytherapy was most frequently radiograph-based using standard plans prescribing to point A and delivered with intracavitary applicators using high dose-rates. The use of interstitial needles and magnetic resonance imaging/computed tomography-based planning were quite uncommon. The cumulative (external beam radiotherapy + brachytherapy) prescribed EQD2 dose range was 78 Gy (95%CI: 76–81 Gy) to 88 Gy (95%CI: 87–90 Gy). Radiotherapy was completed in 91% (95%CI: 90–93%) in patients treated with chemoradiation followed by adjuvant systemic therapy and in 94% (95%CI: 93–95%) of patients treated with chemoradiation (*p* = 0.006).

Tables S3–S5 in supplemental data II shows the agents, doses and schedules of concurrent and adjuvant therapies. Concurrent chemotherapy was mainly platinum-based (93%). In 10 studies, a second agent was added, usually a pyrimidine antagonist. Five of the 12 (42%) controlled studies did not use the same agent(s) as concurrent treatment in the control and experimental arm [11,19,23,24,27].

In nine studies, adjuvant systemic therapy consisted of 1–3 cycles cisplatin and a pyrimidine antagonist. This was 5-fluorouracil in five [18,19,22,23,24,35], gemcitabine in four studies [11,20,21,34,37]. In 12 studies, adjuvant systemic therapy was 3–6 cycles of a platinum derivate (carboplatin [17,25,27,29,30,31,32,33,40], cisplatin [49], cisplatin or carboplatin [28] or nedaplatin [32] with a taxane (paclitaxel in 11 [17,25,27,28,29,30,31,32,33,40,50], docetaxel in 1 [49]). The remaining eight studies investigated adjuvant platinum derivates (*N* = 2) [38,47], pyrimidine antagonists as monotherapy (*N* = 2) [26,36], cis- platin-ifosfamide (*N* = 1) [42,43,44,45,46], 5-fluorouracil with interferon and retinoic acid (*N* = 1) [48], ipilimumab [41] and pembrolizumab [39].

### 3.3. Risk of Bias Assessment

**Figure 2:**
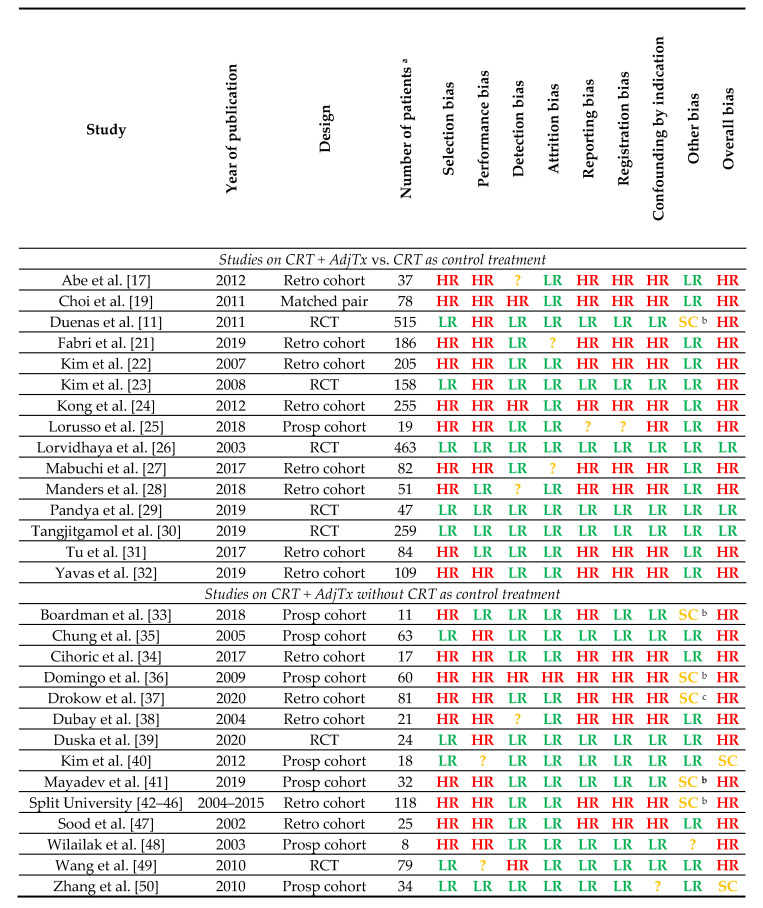
Risk of bias assessment of included studies. Definition of abbreviations: CRT = chemoradiotherapy; AdjTx = adjuvant systemic therapy; RCT = randomized controlled trial; Pros = prospective study; Retro = retrospective study; HR = high risk; LR = low risk; SC = some concerns; ? = unclear risk. ^a^ The number of patients reported in this table are the numbers of patients that could be included in the current study as either a control group (CRT) or experimental group (CRT + AdjTx); patients who were treated with other regimens (e.g., radiotherapy only) are not included in the current study and not represented here. ^b^ Conflicts of interest of one or more authors; relations with or employees of pharmaceutical companies. ^c^ Concerns about correctness of reported survival outcomes; discrepancies between reported estimates and survival curves.

Results of the risk of bias assessment are presented in Figure 2. All studies included in the meta-analyses on adjuvant platinum–pyrimidine antagonist were at high risk of bias. Systematic differences between the study arms leading to the performance bias were present in all six studies. In addition, the retrospective studies were at risk of selection bias, reporting bias, registration bias and confounding by indication. Likewise, five retrospective studies on adjuvant platinum–taxane were classified as at a high risk of bias. Only the randomized controlled trial on adjuvant platinum–taxane by Tangjitgamol et al. was judged to be at low risk of bias [30].

### 3.4. Meta-Analysis

**Figure 3:**
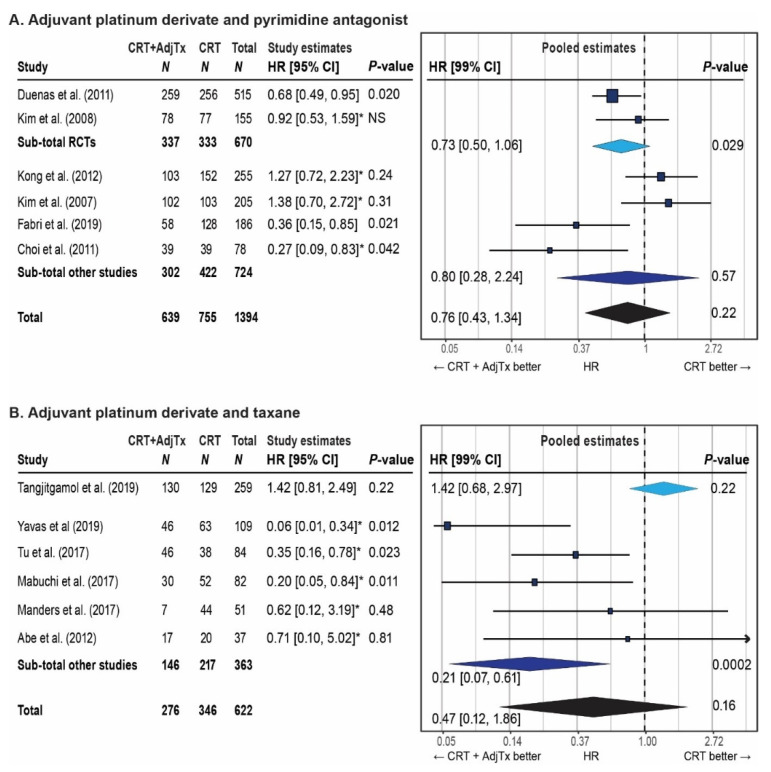
Impact on the overall survival of the addition of adjuvant chemotherapy to chemoradiation and brachytherapy. Each study in the forest plot is represented by a black square which represents the study’s hazard ratio and a whisker on each side that represents the study’s 99% confidence interval. The size of the black square represents the weight of the study in the meta-analysis. The pooled hazard ratios are shown as diamond shapes; the light blue diamond represents the pooled hazard ratio based on only randomized controlled trials, the dark blue diamond represents the pooled hazard ratio based on only non-randomized studies, and the black diamond is the pooled hazard ratio of all studies combined. (**A**) Meta-analysis of overall survival after concurrent chemoradiation and brachytherapy with adjuvant platinum derivate and pyrimidine antagonist vs. concurrent chemoradiation and brachytherapy only. I^2^ = 62%, Q-test *p* = 0.02. (**B**) Meta-analysis of overall survival after concurrent chemoradiation and brachytherapy with adjuvant platinum derivate and taxane vs. concurrent chemoradiation and brachytherapy only. I^2^ = 74%, Q-test *p* = 0.002. Definition of abbreviations: CRT = chemoradiation and brachytherapy; AdjTx = adjuvant therapy; HR = hazard ratio; CI = confidence interval. * Imputed values; methods described in Supplementary Materials I.

**Figure 4:**
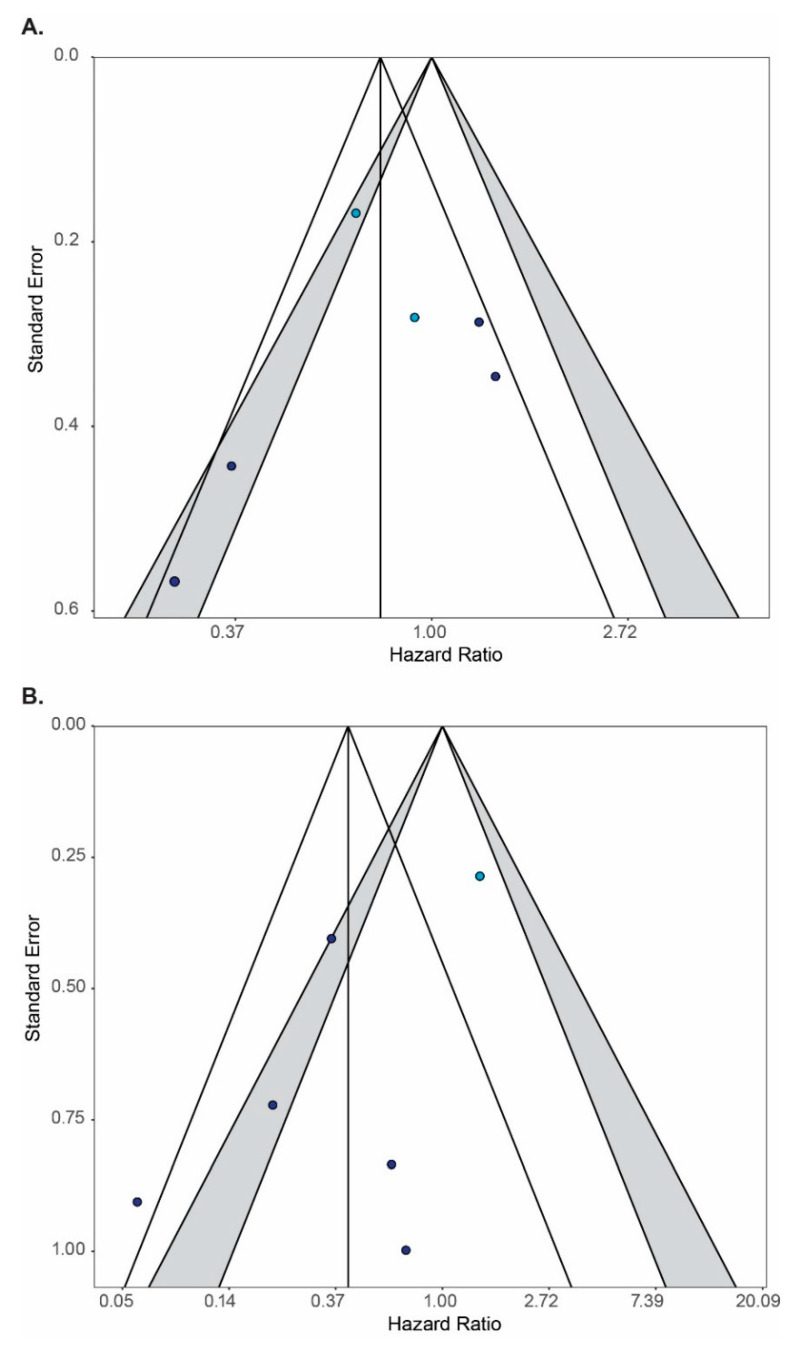
The assessment of the publication bias of studies included in the meta-analysis. Each circle represents a study included in the meta-analysis on overall survival; light blue circles are randomized controlled trials; dark blue circles are non-randomized controlled trials. The grey contours indicate the 95 and 99% confidence intervals of the hazard ratio for the impact of the addition of adjuvant systemic therapy to chemoradiation on the overall survival in the individual studies. The black vertical line is placed at the pooled estimate for the hazard ratio for overall survival based on the studies included in the meta-analysis. The black diagonal lines indicate the 99% confidence interval of the pooled estimate. Lack of symmetry in the presence of studies across the area defined by the black lines may indicate publication bias. Panel (**A**): Funnel plot of the studies on the addition of adjuvant platinum–pyrimidine antagonist after chemoradiation and brachytherapy. Panel (**B**): Funnel plot of the studies on the addition of adjuvant platinum–taxane after chemoradiation and brachytherapy.

Meta-analyses were performed for six studies comparing chemoradiation followed by adjuvant platinum–pyrimidine antagonist with chemoradiation (Figure 3). The pooled hazard ratio estimate for overall survival was 0.76 (99%CI: 0.43–1.34, *p* = 0.22). Heterogeneity (Supplemental Materials II Table S6) and publication bias (Figure 4) are present.

Meta-analyses were also performed for six controlled studies (total *N* = 622) reporting survival outcomes on adjuvant platinum–taxane (Figure 3). The pooled hazard ratio for overall survival was 0.47 (99%CI: 0.12–1.86, *p* = 0.16). Heterogeneity (Supplemental Materials II Table S6) and publication bias (Figure 4) are present. The pooled hazard ratio for recurrence-free survival was 0.68 (99%CI: 0.33-1.41, *p* = 0.17). No meta-analysis could be performed for distant metastasis-free survival as only one study reported on this outcome (HR 0.26, 99%CI: 0.05–1.49, *p* = 0.047) (35).

Sensitivity analysis based on the leave-one-out approach showed that outcomes of the meta-analyses for the primary outcome are robust (Supplemental Materials II Table S7).

### 3.5. Systematic Review of Survival Outcomes

An overview of the survival outcomes reported in all 29 included studies are provided in Tables S8 and S9 of the Supplementary Materials II. Briefly, studies on adjuvant platinum–pyrimidine antagonist after chemoradiation reported 3-year overall survival rates of 70–90% in the experimental arm compared to 69–93% in the control arm. Studies on adjuvant platinum–taxane showed 3-year overall survival rates of 31–80% in the experimental arm compared to 23–93% in the control arm. A randomized controlled trial on adjuvant 5-fluorouracil showed no significant benefit for metastasis-free, recurrence-free and overall survival [26]. A phase I trial on ipilimumab reported a 1-year progression-free survival and overall survival of 81% and 90%, respectively [41].

### 3.6. Systematic Review of Feasibility and Toxicity

The feasibility and severe toxicity of adjuvant systemic therapy are presented Table 3, Table 4 and Table 5. Pooled treatment completion rate was 79% (95%CI: 76–82%) for chemoradiation followed by adjuvant platinum–pyrimidine antagonist and 70% (95%CI: 64–86%) for chemoradiation followed by adjuvant platinum–taxane. Both adjuvant chemotherapy doublets caused more severe acute hematological and gastro-intestinal toxicities than chemoradiation alone. Adjuvant ipilimumab was completed in 86% and immune-mediated toxicity was observed in some patients [41]. Adjuvant pembrolizumab was completed in 100% despite severe gastro-intestinal toxicities and hypothyroidism in 13 and 4%, respectively [39].

## 4. Discussion

This systematic review and meta-analysis evaluated the potential benefit and toxicity of adjuvant systemic therapy after primary chemoradiation and brachytherapy for locally advanced cervical cancer. Twenty-nine studies reporting on adjuvant chemotherapy and immunotherapy were included. The meta-analysis of 12 studies on two chemotherapy doublets (platinum–taxane and platinum–pyrimidine antagonists) showed no significant overall survival benefit while severe acute toxicity was significantly increased. 

In this meta-analysis, two randomized controlled trials on the benefit of the addition of a platinum–pyrimidine antagonist after chemoradiation have been pooled. A significant benefit for distant metastasis-free survival was found. The benefit for overall survival (HR 0.73 95%CI: 0.50–1.06) did not reach statistical significance (*p* = 0.029, predefined as *p* < 0.01 in this meta-analysis). A lack of power could be the reason that this result did not reach significance. The addition of four non-randomized studies, which increased the numbers of patients from 670 to 1394, and did not result in a statistically significant overall survival benefit either (HR 0.76, 95%CI: 0.43–1.34, *p* = 0.22). These pooled estimates should be interpreted with caution, because several forms of bias may have affected trial outcomes. In both randomized controlled trials, the superior outcomes in the experimental arm may have partly been due to the addition of a pyrimidine antagonist to concurrent chemotherapy. In the non-randomized studies, the risk of recurrence and death may not have been in same between study arms. In addition, publication bias is probably present which may have biased the pooled overall survival estimate in favor of adjuvant platinum–pyrimidine antagonist. Clearly, there is a need for a high quality randomized controlled trial that is powered to demonstrate the significance of a benefit around HR 0.75. It is not likely that such a trial will be conducted because all studies showed a substantial increase in severe toxicity. Therefore, adjuvant platinum–pyrimidine antagonist cannot be recommended for unselected patients.

Subgroup-analysis of the randomized controlled trial by Duenas-Gonzales et al. showed that patients with Stage III–IVA, tumors ≥5 cm and of non-adenocarcinoma histotype had benefitted the most from adjuvant treatment [20]. The contrary was found in the study by Fabri et al.: stage ≥IIIA had a significantly worse overall survival despite adjuvant platinum–pyrimidine in multivariable analysis [21]. Kim et al. found that adjuvant platinum–pyrimidine and tumor characteristics were not significantly related to overall survival and disease-free survival in univariable analyses [22]. The other studies on adjuvant platinum–pyrimidine did not report subgroup analysis, therefore no pooled estimates could be calculated for patients with additional risk factors. Hence, current evidence is unclear about a possible benefit in high-risk subgroups, but it is clear about the significant increase in severe toxicity.

The second meta-analysis pooled one randomized controlled trial (*N* = 259) with five small retrospective cohorts (*N* = 363) on adjuvant platinum–taxane. The randomized controlled trial was at low risk of bias and showed no benefit of adjuvant platinum–taxane for overall survival and recurrence-free survival. The addition of the five small studies (all at high risk of bias) did not change this conclusion. However, the pooled estimate would change if a new, large study was added. The outcome of the ongoing OUTBACK study (ANZGOG-0902/GOG-0274/RTOG-1174), a large phase III randomized controlled trial on adjuvant carboplatin–paclitaxel, is required for a conclusion on the benefit of adjuvant platinum–taxane [51]. As such, this meta-analysis could serve as an overview of the literature that facilitates the interpretation of the OUTBACK results.

Considering the toxicity profile, targeted therapies might be an attractive alternative to chemotherapy. Only two phase I–II studies on adjuvant immunotherapy have been published so far. The first by Mayadev et al. (2019) was a phase I trial on the anti-cytotoxic T-lymphocyte antigen-4 (CTLA-4) ipilimumab as an adjuvant agent after chemoradiation [41]. This treatment seems feasible (86% completion) but severe immune-mediated toxicities are not uncommon. The reported 1-year progression-free survival of 81% is difficult to interpret as it was not compared to chemoradiation. In metastatic cervical cancer, ipilimumab showed no significant clinical activity as monotherapy [52].

The second study was published in 2020 by Duska et al.: a phase-II randomized controlled trial on concurrent vs. adjuvant pembrolizumab (PD-1 inhibitor). In both settings pembrolizumab was feasible (83% completion) and severe toxicity was limited [39]. No data on efficacy was reported. The efficacy of pembrolizumab has been demonstrated for PD-L1-positive recurrent and metastatic cervical cancer which led to registration for this indication [53]. A phase-III randomized controlled trial (KEYNOTE-A18) on pembrolizumab during and after chemoradiation compared to chemoradiation is ongoing [54].

In addition to the addition of systemic therapies to chemoradiation, the improvement of radiotherapy techniques is also a way to increase tumor control while reducing toxicity. Image-guided adaptive brachytherapy has already been shown as able to do this [4,5,6,7,8], and new evidence on the impact of intensity-modulated radiotherapy and applying strict dose aims and constraints for external beam radiation therapy are expected to be published soon by, respectively, the investigators of the PARCER trial [55,56] and the EMBRACE II study [57].

This study was limited to adjuvant systemic therapy after chemoradiation and brachytherapy because this may be the most feasible option in terms of toxicity. It also allows for patient selection based on response to chemoradiation, which is an independent predictor of overall survival [58]. 

A couple of weaknesses should be considered when interpreting this meta-analysis. Firstly, there is inter-study heterogeneity which could affect the accuracy of the pooled estimates. For example, in a part of the included studies (prophylactic), extended field radiotherapy has been applied, which could have increased the risk of gastro-intestinal toxicity [59,60]. This is not surprising considering the variety of eligible study designs. We accounted for some heterogeneity by using random-effects models and we verified the robustness of the conclusions by sensitivity analysis. Secondly, the quality of the published studies provides suboptimal evidence. These limitations do not affect, but rather support the conclusion that adjuvant systemic therapy does not improve overall survival in unselected patients. Only if the ongoing OUTBACK study will show a substantial significant overall survival benefit, the conclusion of this meta-analysis could change [51].

## 5. Conclusions

The current standard of care for patients with locally advanced cervical cancer is cisplatin-based chemoradiation and brachytherapy. Improvement of overall survival of these patients has plateaued in the last two decades. This systematic review on the efficacy and toxicity of adjuvant systemic therapy gives an overview of current evidence. Few randomized controlled trials have been published and most studies were at considerable risk of bias. A meta-analysis of the two most investigated chemotherapy doublets showed that there was no significant improvement in overall survival while acute toxicity was significantly increased. Most of the studies on other chemotherapeutic agents did not seem to provide a good balance between efficacy and toxicity either. Current evidence on targeted therapies in the adjuvant setting is immature. Future clinical trials should be selective in the allocation of treatment strategy and focus on agents that increase the therapeutic window between efficacy and toxicity.

## Figures and Tables

**Table 1:** Design of the systematic search.

**Patient**	Tumor characteristics: FIGO stage IB–IVA (including metastasis to the para-aortic lymph nodes) cervical cancer of squamous cell carcinoma, adenocarcinoma or adenosquamous carcinoma histotype Study characteristics: randomized controlled trials, non-randomized prospective and retrospective studies
**Intervention**	External beam radiotherapy to the whole pelvis (with or without integrated or sequential boosts or extended field) with concurrent chemotherapy and intracavitary or interstitial brachytherapy followed by adjuvant systemic therapy (e.g., chemotherapy or immuno therapy)
**Control**	External beam radiotherapy to the whole pelvis (with or without integrated or sequential boosts or extended field) with concurrent chemotherapy and intracavitary or interstitial brachytherapy
**Outcomes**	Overall survival, recurrence- or disease-free survival, metastasis-free survival, treatment completion, toxicity
**Exclusion**	Tumor characteristics: persistent or recurrent cervical cancer, distant metastasis Treatment characteristics: primary surgery, neo-adjuvant systemic therapy Publication types: conference abstracts, case-reports, review articles, meta-analyses, editorials, letters to the editor, guidelines, articles published before the year 2000

**Table 2:** Overview of the 29 included studies on adjuvant systemic therapy after chemoradiation and brachytherapy.

Study	Country	Year	Design	Controlled	N ^a^	Age ^b^	Histology	Stage	Pelvic LN	PAO LN
Abe et al. [17]	Japan	2012	Retro	Yes	37	55 (31–72)	SQ	IB–IVA	Yes	Yes
Choi et al. [18,19]	Korea	2007, 2011	m-pair	Yes	78	53 (33–71)	SQ, AC, ASQ	IB–IVA	Yes	No
Duenas et al. [11,20]	Multiple ^c^	2011, 2012	RCT	Yes	515	46 (18–70)	SQ, AC, ASQ	IIB–IVA	NR	No
Fabri et al. [21]	Brazil	2019	Retro	Yes	186	48	SQ, AC	IB–IVA	Yes	Yes
Kim et al. [22]	Korea	2007	Retro	Yes	205	51 (29–75)	SQ, SCC	IB, IIB	Yes	No
Kim et al. [23]	Korea	2008	RCT	Yes	155	58 (34–75)	SQ, AC, ASQ	IIB–IVA	Yes	No
Kong et al. [24]	Korea	2012	Retro	Yes	255	57 (25–87)	SQ, AC, ASQ	IIB–IVA	Yes	NR
Lorusso et al. [25]	Italy	2018	Pros	Yes	19	48 (34–72)	SQ, AC, ASQ	II–IIIA	Yes	Yes
Lorvidhaya et al. [26]	Thailand	2003	RCT	Yes	463	49	SQ, AC, ASQ, SCC	IIB–IVA	Yes	Yes
Mabuchi et al. [27]	Japan	2017	Retro	Yes	82	53 (30–68)	SQ	IIIB–IVA	Yes	NR
Manders et al. [28]	USA	2018	Retro	Yes	51	48 (29–79)	SQ, AC, ASQ	IB–II, IIIB–IVA	Yes	Yes
Pandya et al. [29]	India	2019	RCT	Yes	47	55 (33–70)	SQ, AC	IIB–IVA	Yes	Yes
Tangjitgamol et al. [30]	Thailand	2019	RCT	Yes	259	50 (23–68)	SQ, AC, ASQ	IIB–IVA	Yes	No
Tu et al. [31]	China	2017	Retro	Yes	84	46 (28–69)	SQ, AC, ASQ	IBM IIB–IIIB	No	No
Yavas et al. [32]	Turkey	2019	Retro	Yes	109	53 (29–85)	SQ, AC, ASQ, SCC, LC	IB–IVA	Yes	Yes
Boardman et al. [33]	USA	2018	Pros	No	10	42 (26–67)	SQ, AC	IB–IVA	Yes	Yes
Cihoric et al. [34]	Switzerland	2017	Retro	No	17	NR	SQ, AC	IB–IVA	Yes	Yes
Chung et al. [35]	Taiwan	2005	Pros	No	63	52 (31–77)	SQ, AC, ASQ	IIB–IVA	Yes	Yes
Domingo et al. [36]	Multiple ^d^	2009	Pros	No	60	47 (28–72)	SQ	IIB–IIIB	NR	NR
Drokow et al. [37]	China	2020	Retro	No	81	45 (25–60)	SQ, AC	IB2–IIIB	Yes	Yes
Dubay et al. [38]	USA	2004	Retro	No	21	36 (25–72)	SQ	IIB–IVA	NR	NR
Duska et al. [39]	USA	2020	Pros	No	24	49 (28–74)	SQ, AC	IB2–IVA	Yes	Yes
Kim et al. [40]	Korea	2012	Pros	No	18	52 (37–74)	SQ, AC, ASQ	IIB–IVA	Yes	Yes
Mayadev et al. [41]	USA	2019	Pros	No	32	50 (26–61)	SQ, AC, ASQ	IB2–IVA	Yes	Yes
Split University [42,43,44,45,46]	Croatia	2004–2015	Retro	No	118	53 (27–77)	SQ, AC, ASQ	IB–IVA	Yes	No
Sood et al. [47]	USA	2002	Retro	No	25	50 (36–73)	SQ	IB–IIIB	Yes	Yes
Wilailak et al. [48]	Thailand	2003	Pros	No	8	45 (39–60)	SQ	IIIB	Yes	NR
Wang et al. [49]	China	2010	RCT	No	79	52 (42–65)	SQ	IIA–IIIB	NR	NR
Zhang et al. [50]	China	2010	Pros	No	34	47 (35–64)	SQ	IIB–IIIB	Yes	No

Subscript Table 2. Definition of abbreviations: CRT = chemoradiotherapy; AdjTx = adjuvant systemic therapy; LN = lymph node; PAO = para-aortic; RCT = randomized controlled trial; Pros = prospective study; Retro = retrospective study; m-pair = matched pair study; NR = not reported; SQ = squamous cell carcinoma; AC = adenocarcinoma; ASQ; adenosquamous carcinoma; SCC = small cell carcinoma; LC = large cell carcinoma. ^a^ The number of patients reported in this table represents the number of patients that could be included in the current study as either a control group (CRT) or experimental group (CRT + AdjTx); patients who were treated with other regimens (e.g., radiotherapy only) are not included in the current study and not represented here. ^b^ Age reported as median (range), or if unavailable, mean (Lorvidhaya et al. [26], Pandya et al. [29], Tu et al. [31], Boardman et al. [33] and Duska et al. [39]). ^c^ Mexico, Argentina, India, Panama, Bosnia Herzegovina, Peru, Thailand, Pakistan, Australia. ^d^ Philippines, Thailand, Australia.

**Table 3:** Feasibility and toxicity of adjuvant platinum derivate and pyrimidine antagonist.

Study	Treatment Arm	*N*	Completion Rate	Severe Acute Toxicity								Severe Late Toxicity	
				Anemia	Leucopenia	Thrombopenia	GI	GU	Neuropathy	Liver	Renal	GI	GU
*Studies with CRT as control treatment*													
Choi et al. [19]	CRT	39	95%	4%	5%	2%	10%	.	0%	0%	0%	0%	0%
	CRT+AdjTx	39	90%	8%	11%	2%	9%	.	0%	0%	0%	0%	0%
Duenas et al. [11]	CRT	256	.	2%	12%	1%	8%	.	.	0%	1%	0%	0%
	CRT+AdjTx	259	77%	9%	51%	6%	26%	.	.	1%	2%	2%	1%
Fabri et al. [21]	CRT	128	.	.	.	.	.	.	.	.	.	.	.
	CRT+AdjTx	58	91%	.	.	.	.	.	.	.	.	.	.
Kim et al. [22]	CRT	103	.	3%	42%	11%	12%	.	.	2%	4%	0%	1%
	CRT+AdjTx	102	63%	12%	77%	13%	23%	.	.	7%	8%	8%	3%
Kim et al. [23]	CRT	77	73%	Any hemat 25%			0%	0%	.	.	.	4%	3%
	CRT+AdjTx	78	65%	Any hemat 41%			8%	3%	.	.	.	1%	0%
Kong et al. [24]	CRT	152	100%	2%	5%	1%	9%	.	.	.	.	.	.
	CRT+AdjTx	103	100%	7%	11%	4%	25%	.	.	.	.	.	.
SUBTOTAL	CRT	755	92%	2%	15%	3%	8%	0%	0%	1%	2%	1%	1%
	95%CI		88–95%	1–4%	12–18%	2–4%	6–10%	0–0%	0–0%	0–1%	0–3%	0–1%	0–1%
	CRT+AdjTx	639	79%	9%	45%	7%	22%	3%	0%	2%	3%	3%	1%
	95%CI		76–82%	7–12%	41–49%	5–9%	18–25%	0–7%	0–0%	1–4%	2–5%	1–4%	0–2%
	*p*-value	1394	<0.0001	<0.0001	<0.0001	0.004	<0.0001	0.5	NS	0.037	0.26	0.012	0.51
*Studies without CRT as control treatment*													
Cihoric et al. [34]	CRT+AdjTx	17	53%	.	.	.	18%	0%	.	.	.	0%	12%
Chung et al. [35]	CRT+AdjTx	63	92%	3%	10%	2%	2%	.	.	.	.	6%	.
Drokow et al. [37]	CRT+AdjTx	81	100%	0%	0%	0%	3%	0%	.	.	.	0%	0%
Wilailak et al. [48]	CRT+AdjTx	8	75%	0%	38%	0%	26%	.	.	.	.	.	.
TOTAL	CRT	755	92%	2%	15%	3%	8%	0%	0%	1%	2%	1%	1%
	95%CI		88–95%	1–4%	12–18%	2–4%	6–10%	0–0%	0–0%	0–1%	0–3%	0–1%	0–1%
	CRT+AdjTx	808	82%	7%	36%	5%	18%	1%	0%	2%	3%	3%	1%
	95%CI		76–87%	3–11%	28–44%	2–9%	12–24%	0–4%	0–0%	1–4%	2–5%	0–5%	0–4%
	*p*-value	1563	<0.0001	<0.0001	<0.0001	0.044	<0.0001	1	NS	0.037	0.26	0.007	0.36

Definition of abbreviations: . = not reported; GI = gastrointestinal; GU = genito–urinal; hemat = hematological; CRT = chemoradiation and brachytherapy; AdjTx = adjuvant systemic therapy. The sub-analysis by study design showed no significant benefit of adjuvant platinum–pyrimidine antagonist in either the randomized controlled trials (HR 0.73, 99%CI: Subscript Table 3 continued: 0.50–1.06, *p* = 0.029) or the non-randomized controlled trials (HR 0.80, 99%CI: 0.28–2.24, *p* = 0.57). The pooled hazard ratio for recurrence-free survival was 0.73 (99%CI: 0.51–1.05, *p* = 0.026) and for distant metastasis-free survival 0.44 (99%CI: 0.25–0.78, *p* = 0.0002).

**Table 4:** Feasibility and toxicity adjuvant platinum derivate and taxane.

Study	Treatment Arm	*N*	Completion	Severe Acute Toxicity							Severe Late Toxicity
				Anemia	Leucopenia	Thrombopenia	GI	GU	Neuropathy	GI	GU
*Studies with CRT as control treatment*											
Abe et al. [17]	CRT	20	.	.	.	.	.	.	.	5%	.
	CRT+AdjTx	17	95%	41%	94%	18%	.	.		6%	.
Lorusso et al. [25]	CRT	9	.	.	.	.	.	.	.	.	.
	CRT+AdjTx	10	90%	10%	.	.	.	.		.	.
Mabuchi et al. [27]	CRT	52	.	.	.	.	.	.	.	.	.
	CRT+AdjTx	30	63%	3%	57%	3%	10%	.	0%	13%	10%
Manders et al. [28]	CRT	44	100%	Any acute hematological tox 11%			5%	.	0%	3%	.
	CRT+AdjTx	7	86%	Any acute hematological tox 0%			0%	.	0%	0%	.
Pandya et al. [29]	CRT	23	70%	17%	17%	.	4%	8%	0%	4%	13%
	CRT+AdjTx	24	79%	13%	33%	.	17%	0%	8%	0%	4%
Tangjitgamol et al. [30]	CRT	129	95%	3%	0%	6%	2%	2%	.	.	.
	CRT+AdjTx	130	65%	5%	13%	4%	5%	3%	3%	.	.
Tu et al. [31]	CRT	38	100%	Any acute hematological tox 24%			13%	.	.	.	.
	CRT+AdjTx	46	.	Any acute hematological tox 37%			11%	.	.	.	.
Yavas et al. [32]	CRT	63	100%	0%	0%	0%	0%	0%	0%	3%	0%
	CRT+AdjTx	46	.	0%	13%	4%	4%	0%	4%	2%	0%
	CRT	378	96%	4%	2%	4%	4%	2%	0%	3%	3%
SUBTOTAL	95%CI		93–98%	1–6%	0–4%	1–7%	1–6%	0–4%	0–0%	1–6%	0–7%
	CRT+AdjTx	310	70%	19%	26%	5%	9%	2%	3%	5%	4%
	95%CI		64–76%	14–23%	20–31%	2–8%	5–12%	0–4%	1–5%	1–8%	0–8%
	*p*-value	688	<0.0001	<0.0001	<0.0001	0.82	0.022	1	0.055	0.55	1
*Studies without CRT as control treatment*											
Boardman et al. [33]	CRT+AdjTx	10	67%	44%	89%	22%	.	.	11%	Any late tox 0%	
Kim et al. [40]	CRT+AdjTx	18	100%	.	15%	.	0%	.	.	6%	0%
Wang et al. [49]	CRT+AdjTx	79	.	48%	58%	25%	63%	.	.	.	.
Zhang et al. [50]	CRT+AdjTx	34	82%	0%	82%	0%	3%	.	0%	6%	3%
	CRT	378	96%	4%	2%	4%	4%	2%	0%	3%	3%
TOTAL	95%CI		93–98%	1–6%	0–4%	1–7%	1–6%	0–4%	0–0%	1–6%	0–7%
	CRT+AdjTx	451	74%	24%	38%	19%	19%	2%	4%	5%	3%
	95%CI		63–85%	16–32%	30–46%	12–26%	12–26%	0–4%	0–10%	0–11%	0–8%
	*p*-value	829	<0.0001	<0.0001	<0.0001	0.027	<0.0001	1	0.063	0.59	1

Subscript Table 4. Definition of abbreviations: . = not reported; GI = gastrointestinal; GU = genito–urinal; hemat = hematological; CRT = chemoradiation and brachytherapy; AdjTx = adjuvant systemic therapy.

**Table 5:** Feasibility and severe toxicity of other adjuvant systemic therapies.

Study	Treatment Arm	*N*	Completion	Severe Acute Toxicity							Severe Late Toxicity	
				Anemia	Leucopenia	Thrombopenia	GI	GU	Renal	Other	GI	GU
Adjuvant cisplatin + ifosfamide												
Split University [42,43,44,45,46]	CRT+AdjTx	118	41%	7%	34%	15%	12%	.	3%	.	Any late tox 19%	
Adjuvant cisplatin												
Dubay et al. [38]	CRT+AdjTx	21	62%	10%	10%	0%	5%	.	0%	.	.	.
Adjuvant carboplatin												
Sood et al. [47]	CRT+AdjTx	25	.	Any acute hematological tox 80%			Any acute non-hemat tox 28%				4%	.
Adjuvant 5-fluorouracil												
Lorvidhaya et al. [26]	CRT	233	95%	0%	4%	2%	.	.	.	.	Any late tox 3%	
	CRT+AdjTx	230	92%	0%	3%	1%	.	.	.	.	Any late tox 6%	
Adjuvant capecitabin												
Domingo et al. [36]	CRT+AdjTx	60	90%	5%	.	.	3%	2%	3%	3% ^a^	.	.
Adjuvant cisplatin + 5-fluorouracil + interferon alpha + retinoic acid												
Wilailak et al. [48]	CRT+AdjTx	8	75%	0%	38%	0%	25%	.	38%	.	.	.
Adjuvant ipilimumab												
Mayadev et al. [41]	CRT+AdjTx	21 ^b^	86%	10%	5%	5%	14%	10%	.	20% ^c^	.	.
Adjuvant pembrolizumab												
Duska et al. [39]	CRT+AdjTx	24	100%	17%	33%	0%	13%	.	.	22% ^d^	.	.

Definition of abbreviations: . = not reported; GI = gastrointestinal; GU = genito–urinal; hemat = hematological; CRT = chemoradiation and brachytherapy; AdjTx = adjuvant systemic therapy. ^a^ Other severe toxicity adjuvant capecitabine: 3% hand–foot syndrome ^b^ 21 of 32 participants were included in the toxicity analysis, remaining patients did not receive ipilimumab. Acute and late toxicities are not reported separately, hence the toxicities displayed in the table occurred at some point during the median follow-up of 15 months. ^c^ Other severe toxicities of adjuvant ipilimumab: 5% lipase increased; 5% cognitive disturbance; 10% skin/subcutaneous ^d^ Other severe toxicities of adjuvant pembrolizumab: 4% hypothyroidism; 4% electrolyte disturbance; 4% syncope.If the OUTBACK trial will show a significant overall survival benefit, it should be large enough to outweigh the negative outcome of the randomized controlled trial by Tangjitgamol et al. [30] and the burden of increased toxicity, to change clinical practice. If the outcome of the OUTBACK trial is negative, then all published studies will agree that adjuvant platinum–taxane is not a good strategy to improve the survival of patients with locally advanced cervical cancer.

## Data Availability

Data are available upon reasonable request.

## Supplementary Materials

The following are available online at https://www.mdpi.com/article/10.3390/cancers13081880/s1, Supplemental Materials I contains: search terms PubMed, EMBRASE and Web of Science; data extraction details; risk of bias assessment protocol; supplementary statistical methods; details on statistical software. Supplementary Materials II contains: Table S1a: Overview of radiotherapy techniques in the 29 included studies (1/2); Table S2: Overview of radiotherapy techniques in the 29 included studies (2/2); Table S3: Concurrent and adjuvant strategies in studies on adjuvant platinum derivate and pyrimidine antagonist (1/3); Table S4: Concurrent and adjuvant strategies in studies on adjuvant platinum derivate and taxane (2/3); Table S5: Concurrent and adjuvant strategies in studies on other adjuvant therapies (3/3); Table S6: Meta-analysis of survival outcomes and post hoc analysis; Table S7: Sensitivity analysis of survival outcomes; Table S8. Survival outcomes (1/2); Table S9. Survival outcomes (2/2).
