# Supplementary material for: Adjuvant Systemic Therapy after Chemoradiation and Brachytherapy for Locally Advanced Cervical Cancer: A Systematic Review and Meta-Analysis
Source: Cancers (Basel). 2021 Apr 14;13(8):1880. doi: 10.3390/cancers13081880 (PMC8070970; doi:10.3390/cancers13081880)
Supplement: Supplementary file 1 [file cancers-13-01880-s001.zip › Cancers-1149973_supplementary/cancers-1149973_supplementary II_XML.docx]

Review

Supplementary Materials II: Adjuvant Systemic Therapy after Chemoradiation and Brachytherapy for Locally Advanced Cervical Cancer: A Systematic Review and Meta-Analysis

Nanda Horeweg ^1,^*, Prachi Mittal ^2^, Patrycja L. Gradowska ^3^, Ingrid Boere ^4^, Supriya Chopra ^5,†^ and Remi A. Nout ^6,†^

**Table S1.** Overview of radiotherapy techniques in the 29 included studies (1/2).

| **First author** |  | **External Beam Radiotherapy** | | | | | | | | | | | | | | | |
| --- | --- | --- | --- | --- | --- | --- | --- | --- | --- | --- | --- | --- | --- | --- | --- | --- | --- |
|  | **Year** | **Delivery technique** | **Imaging technique** | **Planning**  **technique** | **Boost for pos ln** | **Extended field for pos ln*** | **Prophylactic extended field*** | **Dose to tumor in Gy** | | | **Dose to pelvis in Gy** | | **Dose to ln including boost in Gy** | **Dose to PAO ln in Gy** | | |  |
|  |  |  |  |  |  |  |  | **total** | | **fraction** | **total** | **fraction** |  |  | | |  |
| Studies with a control group of chemoradiation and brachytherapy | | | | | | | | | | | | | | | | | |
| Abe(28) | 2012 | APPA, Box | . | 3D | Yes | Yes | No | 20-40 | 2.0 | | 50 | 2.0 | 51-55 | | 51-55 | |  |
| Choi(17) | 2011 | Box | CT, MRI | 3D | . | No | No | 39.6-45 | 1.8 | | 50.4 | 1.8 | 50.4 | | NA | |  |
| Duenas-Gonzáles(11) | 2012 | APPA, Box | . | . | . | No | No | 50.4 | 1.8 | | 50.4 | 1.8 | 50.4 | | NA | |  |
| Fabri(27) | 2019 | . | . | 2D, 3D | Yes | Yes | . | 50.4 | 1.8 | | 50.4 | 1.8 | 54 | | 45 | |  |
| Kim(23) | 2007 | . | . | 2D | . | No | No | 27-36 | 1.8 | | 45-50.4 | 1.8 | 45-50.4 | | NA | |  |
| Kim(18) | 2008 | Box | CT | 2D | . | No | No | 41.1-50.4 | 1.8 | | 41.4-50.4 | 1.8 | 41.4-50.4 | | NA | |  |
| Kong(19) | 2012 | Box | . | 2D | . | Yes | No | 45 | 1.8 | | 45 | 1.8 | 45 | | 45 | |  |
| Lorusso(31) | 2018 | VMAT | CT | 3D | Yes | Yes | No | 50.4 | 1.8 | | 50.4 | 1.8 | 56 | | 45 | |  |
| Lorvidhaya(41) | 2003 | APPA, Box | X-ray | 2D | No | No | No | 40-50 | 2.0 | | 50 | 2.0 | 50 | | NA | |  |
| Mabuchi(20) | 2017 | Box | CT | 3D | . | No | No | 30-40 | 2.0 | | 50 | 2.0 | 50 | | NA | |  |
| Manders(37) | 2018 | Box, IMRT | CT | 3D | . | Yes | NA | >45 | . | | >45 | . | >45 | | 36-59.7 | |  |
| Pandya(32) | 2019 | . | . | . | . | Yes | No | 50.4 | 1.8 | | 50.4 | 1.8 | . | | 45 | |  |
| Tangjitgamol(33) | 2019 | APPA, Box | . | . | No | No | No | 45-50.4 | 1.8-2.0 | | 45-50.4 | 1.8-2.0 | 45-50.4 | | NA | |  |
| Tu(34) | 2017 | APPA | X-ray | 2D | No | No | No | 30 | 2.0 | | 50 | 2.0 | 50 | | NA | |  |
| Yavas(35) | 2019 | APPA, Box | . | 3D | No | Yes | No | 45 | 1.8 | | 50.4 | 1.8 | 50.4 | | 45 | |  |
| Studies without a control group of chemoradiation and brachytherapy | | | | | | | | | | | | | | | | | |
| Boardman(29) | 2018 | . | . | . | . | Yes | Yes | 45 | 1.5 | | 45 | 1.5 | 45 | | | 45 |  |
| Cihoric(24) | 2017 | . | (PET-)CT, MRI | 3D | . | . | . | 50.4-54 | 1.8 | | 50.4 | 1.8 | 50.4 | | | . |  |
| Chung(22) | 2005 | Box | CT | 3D | Yes | Yes | Yes | 45 | 1.8 | | 50.4 | 1.8 | 50.4-65.4 | | | 45 |  |
| Domingo(42) | 2009 | . | . | . | . | . | . | 45 | 1.8 | | 45 | 1.8 | 45 | | | . |  |
| Drokow(25) | 2020 | IMRT | (PET-)CT, MRI | 3D | . | . | No | 50.4-54.0 | 1.8 | | 50.4 | 1.8 | . | | | . |  |
| Dubay(39) | 2004 | . | . | . | . | . | . | 50.4 | 1.8 | | 50.4 | 1.8 | 50.4 | | | . |  |
| Duska(50) | 2020 | IMRT, VMAT | . | 3D | Yes | Yes | Yes | 45 | 2.0 | | 45 | 2.0 | 63 | | | 63 |  |
| Kim(30) | 2012 | APPA, Box | . | . | . | Yes | Yes | 50-61.2 | 1.8-2.0 | | 50-61.2 | 1.8-2.0 | 50-61.2 | | | 45-63 |  |
| Mayadev(49) | 2019 | No IMRT | . | . | . | Yes | Yes | 45 | 1.8 | | 45 | 1.8 | 45 | | | 45 |  |
| Split University(43-47) | 2004-2015 | APPA, Box | X-ray, CT | 2D, 3D | . | No | No | 25 | 2.0 | | 50 | 2.0 | 50 | | | NA |  |
| Sood(40) | 2002 | Box | . | . | . | Yes | . | 50.4-59.4 | 1.8 | | 50.4-59.4 | 1.8 | 50.4-59.4 | | | 45 |  |
| Wilailak(48) | 2003 | APPA, Box | . | . | . | . | . | 45-50.4 | 1.8 | | 45-50.4 | 1.8 | 45-50.4 | | | . |  |
| Wang(36) | 2010 | APPA, Box | . | . | . | . | . | 46-50 | 2.0 | | 46-50 | 2.0 | 46-50 | | | . |  |
| Zhang(38) | 2010 | APPA | X-ray | 2D | No | No | No | 30 | 1.8 | | 45-50.4 | 1.8 | 45-50.4 | | | NA |  |

Definition of abbreviations: EBRT = external beam radiotherapy; CRT = chemoradiotherapy; Adj Tx = adjuvant therapy; PAO = para-aortal; ln = lymph node; APPA = 2 opposing fields; Box = 4 fields box technique; IMRT = intensity-modulated radiotherapy; VMAT = volumetric arc technique; CT = computed tomography; MRI = magnetic resonance imaging; PET = positron emission tomography; D = dimensional; # = fractions; . = not reported; NA = not applicable * External beam radiotherapy fields are extended to include the para-aortic lymph nodes.

**Table S2.** Overview of radiotherapy techniques in the 29 included studies (2/2).

| **Study** |  | **Brachytherapy** | | | | | | | **External Beam Radiotherapy + Brachytherapy** | | | |  |
| --- | --- | --- | --- | --- | --- | --- | --- | --- | --- | --- | --- | --- | --- |
|  | **Year** | **Indication for BT in all patients** | **Application** | **Dose tempo** | **Imaging technique** | **Planning**  **technique** | **Dose to target in Gy** | | **Cumulative target EQD2 (EBRT + brachy)** | **Completion in CRT +**  **AdjTx group** | **Completion in CRT only group** |  |  |
|  |  |  |  |  |  |  | total | fraction |  |  |  |  |  |
| Studies with a control group of chemoradiation and brachytherapy | | | | | | | | | | | | | |
| Abe et al.(28) | 2012 | Yes | IC | HDR | . | 2D | 15-24 | . | . | 100% | 100% |  |  |
| Choi et al.(17) | 2011 | Yes | IC | HDR | . | 2D | 24 | 4.0 | 66.9-72.3 Gy | 100% | . |  |  |
| Duenas-Gonzáles et al.(11) | 2011 | Yes | IC | LDR, MDR | . | 2D | 30-35 | . | . | 94.5% | 91.9% |  |  |
| Fabri et al.(27) | 2019 | Yes | . | HDR | . | 2D | 28 | 7.0 | 89.2 Gy | 85.9% | 94.8% |  |  |
| Kim et al.(23) | 2007 | Yes | IC | HDR | . | 2D | 30 | 5.0 | 64.1-72.9 Gy | . | . |  |  |
| Kim et al.(18) | 2008 | Yes | IC | HDR | . | 2D | 30-35 | 5.0 | 77.9-93.3 Gy | 89.6% | 76.9% |  |  |
| Kong et al.(19) | 2012 | Yes | IC | . | . | 2D | 30 | 6.0 | 84.3 Gy | 92.8% | 86.4% |  |  |
| Lorusso et al.(31) | 2018 | Yes | IC | HDR | CT, MRI | 3D | 25 | 5.0 | 80.8 Gy | 100% | 100% |  |  |
| Lorvidhaya et al.(41) | 2003 | Yes | IC | MDR, HDR | . | 2D | 28-30 | 7.0-7.5 | 79.7-93.8 Gy | . | . |  |  |
| Mabuchi et al.(20) | 2017 | Yes | IC, IC+IS | HDR | CT | 3D | 27.2 | 6.8 | 68.1-78.1 Gy | . | . |  |  |
| Manders et al.(37) | 2018 | Yes | . | . | . | . | . | . | . | . | . |  |  |
| Pandya et al.(32) | 2019 | Yes | IC | HDR | . | . | 21 | 7.0 | 79.3 Gy | 100% | 100% |  |  |
| Tangjitgamol et al.(33) | 2019 | Yes | IC | HDR | . | . | 18-30 | 6.0-7.5 | 68.3-94.2 Gy | 96.1% | 93.8% |  |  |
| Tu et al.(34) | 2017 | Yes | IC | HDR | CT | 2D | 45 | 7.0-10.0 | 93.8-105.0 Gy | . | . |  |  |
| Yavas et al.(35) | 2019 | Yes | IC | HDR | . | 3D | 28 | 7.0 | 83.9 Gy | 100% | 100% |  |  |
| Studies without a control group of chemoradiation and brachytherapy | | | | | | | | | | | | | |
| Boardman et al.(29) | 2018 | Yes | IC | LDR, HDR | . | . | . | . | . | 90.9% | NA |  |  |
| Cihoric et al.(24) | 2017 | Yes | IC | HDR | CT, MRI | 3D | 18-24 | 6.0 | 73.6-85.1 Gy | 100% | NA |  |  |
| Chung et al.(22) | 2005 | Yes | IC | HDR | . | 3D | 22-31 | 4.0-7.0 | 69.9-88.2 Gy | 100% | NA |  |  |
| Domingo et al.(42) | 2009 | Yes | . | HDR | . | . | 26 | 6.5 | 80.0 Gy | 100% | NA |  |  |
| Drokow et al.(25) | 2020 | Yes | IC | HDR | CT. MRI | 3D | 24 | 6.0 | 81.6-85.1 Gy | 100% | NA |  |  |
| Dubay et al.(39) | 2004 | Yes | IC | LDR, HDR | . | 2D | . | . | . | . | NA |  |  |
| Duska et al.(50) | 2020 | Yes | IC | . | . | 3D | . | . | ≥80 Gy | 100% | NA |  |  |
| Kim et al.(30) | 2012 | Yes | IC | LDR | . | . | 22-34.6 | . | . | 100% | NA |  |  |
| Mayadev et al.(49) | 2019 | Yes | IC | LDR, HDR | . | . | 30-40.0 | . | . | 100% | NA |  |  |
| Split University(43-47) | 2004-2015 | Yes | IC | LDR | . | 2D | 60.0 | 30.0 | 85.0 Gy | 100% | NA |  |  |
| Sood et al.(40) | 2002 | Yes | IC+IS | HDR | X-rays | 2D | . | 7.0-11.0 | . | . | NA |  |  |
| Wilailak et al.(48) | 2003 | Yes | IC | HDR | . | 2D | 18-28 | 6.0-7.0 | 68.3-89.2 Gy | 100% | NA |  |  |
| Wang et al.(36) | 2010 | Yes | IC | . | . | 2D | 40-48 | 4.0 | 92.7-106.0 Gy | . | NA |  |  |
| Zhang et al.(38) | 2010 | Yes | IC | HDR | . | 2D | 35-50 | 5.0 | 73.3-92.0 Gy | 100% | NA |  |  |

Definition of abbreviations: EBRT = external beam radiotherapy; CRT = chemoradiotherapy; Adj Tx = adjuvant therapy; PAO = para-aortal; ln = lymph node; APPA = 2 opposing fields; Box = 4 fields box technique; IMRT = intensity-modulated radiotherapy; VMAT = volumetric arc technique; CT = computed tomography; MRI = magnetic resonance imaging; PET = positron emission tomography; D = dimensional; # = fractions; . = not reported; NA = not applicable * External beam radiotherapy fields are extended to include the para-aortic lymph nodes.

**Table S3.** Concurrent and adjuvant strategies in studies on adjuvant platinum derivate and pyrimidine antagonist (1/3).

| Study | Year | Concurrent chemoradiation (CRT) | Concurrent chemoradiation followed by adjuvant systemic therapy (AdjTx) | |
| --- | --- | --- | --- | --- |
|  |  | Agent(s) and schedule of CRT | Agent(s) and schedule of CRT | Agent(s) and schedule of AdjTx |
| Choi et al.(17) | 2011 | 6x Cisplatin 40mg/m^2^ D1 q1w | 3x Cisplatin 60mg/m^2^ D1 q3w  3x 5-FU 1000mg/m^2^ D1-5 q3w | 3x Cisplatin 60mg/m^2^ D1 q3w  3x 5-FU 1000mg/m^2^ D1-5 q3w |
| Cihoric et al.(24) | 2017 | No control arm of CRT | 6x Cisplatin 40mg/m^2^ D1 q1w  6x Gemcitabine 125mg D1 q1w | 2x Cisplatin 50mg/m^2^ D1 q3w  2x Gemcitabine 1000mg/m^2^ D1,8 q3w |
| Chung et al.(22) | 2005 | No control arm of CRT | 2x Cisplatin 50-80mg/m^2^ D1 q3w | 2x Cisplatin 60-80 D1 q1m  2x 5-FU 600-800mg D1-4 q1m |
| Drokow et al.(25) | 2020 | No control arm of CRT | 6x Cisplatin 30mg/m^2^ D1 q1w  6x Gemcitabine 125mg/m^2^ D1 q1w | 2x Cisplatin 25mg/m^2^ D1,2,3 q3w  2x Gemcitabine 1000mg/m^2^ D1,8 q3w |
| Duenas-Gonzáles et al.(11) | 2011 | 6x Cisplatin 40mg/m^2^ D1 q1w | 6x Cisplatin 40mg/m^2^ D1 q1w  6x Gemcitabine 125mg/m^2^ D1 q1w | 2x Cisplatin 50mg/m^2^ D1 q3w,  2x Gemcitabine 1000mg/m^2^ D1,8 q3w |
| Fabri et al.(27) | 2019 | 5x Cisplatin 40mg/m^2^ D1 q1w | 5x Cisplatin 40mg/m^2^ D1 q1w | 2x Cisplatin 50mg/m^2^ D1 q3w  2x Gemcitabine 1000mg/m^2^ D1,8 q3w |
| Kim et al.(23) | 2007 | 3x Cisplatin 100mg/m^2^ D1 q3w  3x 5-FU 1000mg/m^2^ D1-5 q3w | 3x Cisplatin 100mg/m^2^ D1 q3w  3x 5-FU 1000mg/m^2^ D1-5 q3w | 3x Cisplatin 100mg/m^2^ D1 q3w  3x 5-FU 1000mg/m^2^ D1-5 q3w |
| Kim et al.(18) | 2008 | 6x Cisplatin 30mg/m^2^ D1 q1w | 2x Cisplatin 100mg/m^2^ D1 q1m  2x 5-FU 1000 mg/m^2^ D1 q1m | 1x Cisplatin 100mg/m^2^ D1 q1m  1x 5-FU 1000 mg/m^2^ D1 q1m |
| Kong et al.(19) | 2012 | 6x Cisplatin 40-70mg/m^2^ D1 q1w | 2x Cisplatin 70mg/m^2^ D1 q4w  2x 5-FU 1000mg/m^2^ D2-5 q4w | 2x Cisplatin 70mg/m^2^ D1 q4w  2x 5-FU 1000mg/m^2^ D2-5 q4w |

Definition of abbreviations: CRT = chemoradiotherapy; AdjTx = adjuvant systemic therapy; D1 = day 1; q3w = cycle of 3 weeks; 5-FU = 5-Fluorouracil.

**Table S4.** Concurrent and adjuvant strategies in studies on adjuvant platinum derivate and taxane(2/3).

| Study | Year | Concurrent chemoradiation (CRT) | Concurrent chemoradiation followed by adjuvant systemic therapy (AdjTx) | |
| --- | --- | --- | --- | --- |
|  |  | Agent(s) and schedule of CRT | Agent(s) and schedule of CRT | Agent(s) and schedule of AdjTx |
| Abe et al.(28) | 2012 | 5x Cisplatin 30mg/m^2^ D1 q1w | 5x Cisplatin 30mg/m^2^ D1 q1w | 6x Carboplatin AUC 6 D1 q4w  6x Paclitaxel 175mg/m^2^ D1 q4w |
| Boardman et al.(29) | 2018 | No control arm of CRT | 6x Cisplatin 40mg/m^2^ D1 q1w | 4x Carboplatin AUC 4-5 D1 q3w  4x Paclitaxel 135mg/m^2^ D1 q3w |
| Kim et al.(30) | 2012 | No control arm of CRT | 3x Carboplatin AUC 5 D1 q3w  3x Paclitaxel 135mg/m^2^ D1 q3w | 3x Carboplatin AUC 5 D1 q3w  3x Paclitaxel 175mg/m^2^ D1 q3w |
| Lorusso et al.(31) | 2018 | 5x Cisplatin 40mg/m^2^ D1 q1w  5x Paclitaxel D1 q1w (dose NR) | 5x Cisplatin 40mg/m^2^ D1 q1w  5x Paclitaxel D1 q1w (dose NR) | 4x Carboplatin AUC 5 D1 q3w  3x Paclitaxel 175mg/mg D1 q3w |
| Mabuchi et al.(20) | 2017 | 5x Nedaplatin 40mg/m^2^ D1 q1w | 5x Carboplatin AUC 2 D1 q1w  5x Paclitaxel 35mg/m^2^ D1 q1w | 3x Carboplatin AUC 5 D1 q3-4w  3x Paclitaxel 175mg/m^2^ D1 q3-4w |
| Manders et al.(37) | 2018 | 5x Cisplatin 40mg/m^2^ D1 q1w | 5x Cisplatin 40mg/m^2^ D1 q1w | 4-6x Cis/Carboplatin 50mg/m^2^/AUC 5  4-6x Paclitaxel 175mg/m^2^ |
| Pandya et al.(32) | 2019 | 5-6x Cisplatin 40mg/m^2^ D1 q1w | 5-6x Cisplatin 40mg/m^2^ D1 q1w | 3x Carboplatin AUC 5 D1 q3w  3x Paclitaxel 155mg/m^2^ D1 q3w |
| Tangjitgamol et al.(33) | 2019 | 6x Cisplatin 40mg/m^2^ D1 q1w | 6x Cisplatin 40mg/m^2^ D1 q1w | 3x Carboplatin AUC 5 D1 q4w  3x Paclitaxel 175mg/mg D1 q4w |
| Tu et al.(34) | 2017 | 3x Carboplatin AUC 4 D2 q3-4w  3x Paclitaxel 135mg/m^2^ D1 q3-4w | 3x Carboplatin AUC 4 D2 q3-4w  3x Paclitaxel 135mg/m^2^ D1 q3-4w | 3x Carboplatin AUC 4 D2 q3-4w  3x Paclitaxel 135mg/m^2^ D1 q3-4w |
| Wang et al.(36) | 2010 | No control arm of CRT | 6x Cisplatin 40mg/m^2^ D1 q1w | 3x Cisplatin 120mg/m^2^ D1 q3w  3x Docetaxel 60mg/m^2^ D1 q3w |
| Yavas et al.(35) | 2019 | 6x Cisplatin 40mg/m^2^ D1 q1w | 6x Cisplatin 40mg/m^2^ D1 q1w | 6x Carboplatin AUC 5 D1  6x Paclitaxel 175mg/m^2^ D1 |
| Zhang et al.(38) | 2010 | No control arm of CRT | 6x Nedaplatin 20mg/m^2^ D1 q1w  6x Paclitaxel 35mg/m^2^ D1 q1w | 4x Nedaplatin 60mg D1 q3w  4x Paclitaxel 135mg/m^2^ D1 q3w |

Definition of abbreviations: CRT = chemoradiotherapy; AdjTx = adjuvant systemic therapy; NR = not reported; D1 = day 1; q3w = cycle of 3 weeks.

**Table S5.** Concurrent and adjuvant strategies in studies on other adjuvant therapies (3/3).

| Study | Year | Concurrent chemoradiation (CRT) | Concurrent chemoradiation followed by adjuvant systemic therapy (AdjTx) | |
| --- | --- | --- | --- | --- |
|  |  | Agent(s) and schedule of CRT | Agent(s) and schedule of CRT | Agent(s) and schedule of AdjTx |
| Split University(43-47) | 2004-  2015 | No control arm of CRT | 2x Cisplatin 75mg/m^2^ D1 q3w  2x Ifosfamide 2000mg/m^2^ D1 q3w | 2x Cisplatin 75mg/m^2^ D1 q3w  2x Ifosfamide 2000mg/m^2^ D1-3 q3w |
| Dubay et al.(39) | 2004 | No control arm of CRT | 3x Carboplatin AUC 4 D1 q3w | 3x Carboplatin AUC 4 D1 q3w |
| Sood et al.(40) | 2002 | No control arm of CRT | 2x Cisplatin 20mg/m^2^ D1-5 q3w | 1x Cisplatin 20mg/m^2^ D1-5 q3w |
| Lorvidhaya(41) | 2003 | Mitomycin C 10mg/m^2^ D1, D29,  5-FU 300mg D1-14, D29-42 | Mitomycin C 10mg/m^2^ D1, D29  5-FU 300mg D1-14, D29 | 3x 5-FU 200mg D1-28 q6w |
| Domingo et al.(42) | 2009 | No control arm of CRT | 8x Capecitabine 1650mg/m^2^ D1-5 q1w | 6x Capecitabine 2000mg D1-14 q3w |
| Wilailak et al.(48) | 2003 | No control arm of CRT | 8x Cisplatin 10-20mg/m^2^ D1 q1w  8x 5-FU 500mg/m^2^ D1 q1w  8x IFα-2a D1,3,5 q1w  8x RA 0.5mg/kg D1-7 q1w | 4x Cisplatin 10-20mg/m^2^ D1 q1w  4x 5-FU 500mg/m^2^ D1 q1w  4x IFα-2a D1,3,5 q1w  4x RA 0.5mg/kg D1-7 q1w |
| Mayadev et al.(49) | 2019 | No control arm of CRT | 6x Cisplatin 40mg/m^2^ D1 q1w | 4x Ipilimumab 3-10mg D1 q3w |
| Duska et al.(50) | 2020 | No control arm of CRT | 5-6x Cisplatin 40mg/m^2^ D1 q1w | 3x Pembrolizumab 200mg D1 q3w |

Definition of abbreviations: CRT = chemoradiotherapy; AdjTx = adjuvant systemic therapy; D1 = day 1; q3w = cycle of 3 weeks; 5-FU = 5-Fluorouracil; IFα-2a = interferon alpha-2a; RA = 13-cis-retinoic acid.

**Table S6.** Meta-analysis of survival outcomes and post hoc analysis.

| Studies | Pooled estimates meta-analysis | | | Post hoc analysis of heterogeneity | |
| --- | --- | --- | --- | --- | --- |
|  | HR | 99%CI | p-value | I^2^ | Q test p-value |
| Studies on adjuvant platinum derivate and pyrimidine antagonist | | | | | |
| Overall survival | | | | | |
| RCTs | 0.73 | 0.50-1.06 | 0.029 | 0% | 0.36 |
| Non-RCTs | 0.80 | 0.28-2.24 | 0.57 | 74% | 0.008 |
| All | 0.76 | 0.43-1.34 | 0.22 | 62% | 0.022 |
| Recurrence-free survival | | | | | |
| RCTs | 0.74 | 0.47-1.17 | 0.088 | 23% | 0.25 |
| Non-RCTs | 0.72 | 0.39-1.33 | 0.17 | 49% | 0.12 |
| All | 0.73 | 0.51-1.05 | 0.026 | 31% | 0.20 |
| Distant metastasis-free survival | | | | | |
| RCTs | 0.35 | 0.17-0.69 | <0.0001 | 0% | 1.00 |
| Non-RCTs | 0.57 | 0.24-1.38 | 0.10 | 25% | 0.26 |
| All | 0.44 | 0.25-0.78 | 0.0002 | 15% | 0.32 |
| Studies on adjuvant platinum derivate and taxane | | | | | |
| Overall survival | | | | | |
| RCTs | 1.42 | 0.68-2.97 | 0.22 | 0% | 1.00 |
| Non-RCTs | 0.21 | 0.07-0.61 | 0.0002 | 25% | 0.26 |
| All | 0.47 | 0.12-1.86 | 0.16 | 74% | 0.002 |
| Recurrence-free survival | | | | | |
| RCTs | 1.26 | 0.71-2.23 | 0.30 | 0% | 1.00 |
| Non-RCTs | 0.44 | 0.21-0.89 | 0.003 | 0% | 0.48 |
| All | 0.68 | 0.33-1.41 | 0.17 | 50% | 0.075 |

Definition of abbreviations: HR = hazard ratio; CI = confidence interval; RCT = randomized controlled trial. The threshold for the presence of significant statistical heterogeneity between studies was defined as an I^2^ value >50% and a p-value for the Q statistics <0.05. **Table S7.** Sensitivity analysis of survival outcomes.

| Study | OS | | RFS | | DMFS | |  |
| --- | --- | --- | --- | --- | --- | --- | --- |
|  | HR | 99%CI | HR | 99%CI | HR | 99%CI |  |
| Studies on adjuvant platinum derivate and pyrimidine antagonist | | | | | | | |
| Duenas-Gonzalez et al.(11) | 0.78 | 0.38-1.59 | 0.75 | 0.47-1.21 | 0.50 | 0.21-1.17 |  |
| Kim et al.(18) | 0.73 | 0.37-1.42 | 0.70 | 0.47-1.03 |  |  |  |
| Kong et al.(19) | 0.69 | 0.38-1.24 | 0.69 | 0.47-1.00 |  |  |  |
| Kim et al.(23) | 0.69 | 0.39-1.22 | 0.71 | 0.47-1.07 | 0.36 | 0.21-0.61 |  |
| Fabri et al.(27) | 0.86 | 0.51-1.47 | 0.79 | 0.59-1.06 | 0.46 | 0.19-1.12 |  |
| Choi et al.(17) | 0.86 | 0.52-1.42 | 0.76 | 0.52-1.12 | 0.45 | 0.22-0.92 |  |
| Studies on adjuvant platinum derivate and taxane | | | | | | | |
| Tangjitgamol et al.(33) | 0.29 | 0.12-0.75 | 0.56 | 0.32-1.00 |  |  |  |
| Yavas et al.(35) | 0.57 | 0.19-1.74 | 0.83 | 0.48-1.44 |  |  |  |
| Tu et al.(34) | 0.41 | 0.08-2.05 | 0.76 | 0.36-1.59 |  |  |  |
| Mabuchi et al.(20) | 0.47 | 0.12-1.84 | 0.71 | 0.32-1.59 |  |  |  |
| Manders et al.(37) | 0.38 | 0.09-1.59 | 0.68 | 0.32-1.46 |  |  |  |
| Abe et al.(28) | 0.38 | 0.09-1.52 | 0.65 | 0.30-1.41 |  |  |  |

Definition of abbreviations: OS = overall survival; RFS = recurrence-free survival; DMFS = distant metastasis-free survival; HR = hazard ratio; CI = confidence interval;. In this table the results of the sensitivity analysis according to the leave-one-out method are provided. In each row the pooled estimates are provided of meta-analyses including all studies except the study mentioned in that row. These results show to what extend the results of the meta-analysis have been affected by each single study.

**Table S8.** Survival outcomes (1/2).

|  |  |  | Overall survival | | | Recurrence-free survival | | | | Distant metastasis-free survival | | | | Risk of bias |
| --- | --- | --- | --- | --- | --- | --- | --- | --- | --- | --- | --- | --- | --- | --- |
| Study | Study arm | N | 2 yr | 3 yr | p | 2 yr | 3 yr | p | 2 yr | | 3 yr | p |  | |
| Studies on adjuvant platinum derivate and pyrimidine antagonist | | | | | | | | | | | | | | |
| Choi et al.(17) | CRT  CRT+AdjTx | 39  39 | ~88%  ~96% | 70%  93% | 0.042 | ~60%  ~85% | 55%  70% | 0.079 | . | | ~77%  ~92% | 0.060 | HR | |
| Cihoric et al.(24) | CRT+AdjTx | 17 | . | ~82% | . | ~85% | ~59% | . | . | | ~65% | . | HR | |
| Chung et al.(22) | CRT+AdjTx | 63 | . | 81% | . | . | ~81% | . | ~86% | | 81% | . | HR | |
| Drokow et al.(25) | CRT+AdjTx | 81 | . | 75% | . | . | 78% | . | . | | . | . | HR | |
| Duenas-Gonzáles et al.(11) | CRT  CRT+AdjTx | 256  259 | ~75%  ~84% | ~70%  ~80% | 0.020 | ~70%  ~80% | 65%  74% | 0.020 | . | | ~84%  ~92% | 0.005 | HR | |
| Fabri et al.(27) | CRT  CRT+AdjTx | 128  58 | ~86%  ~94% | 81%  91% | 0.021 | ~75%  ~84% | 63%  75% | 0.006 | ~67%  ~87% | | ~63%  ~80% | 0.013 | HR | |
| Kim et al.(23) | CRT  CRT+AdjTx | 103  102 | ~90%  ~85% | ~87%  ~80% | 0.31 | ~87%  ~78% | ~85%  ~78% | 0.45 | . | | ~92%  ~93% | 0.90 | HR | |
| Kim et al.(18) | CRT  CRT+AdjTx | 77  78 | ~75%  ~75% | ~70%  ~69% | NS | ~75%  ~70% | ~65%  ~70% | NS | . | | ~82%  ~79% | NS | HR | |
| Kong et al.(19) | CRT  CRT+AdjTx | 152  103 | ~94%  ~91% | ~90%  ~85% | 0.24 | ~80%  ~78% | ~78%  ~72% | 0.72 | . | | ~86%  ~88% | . | HR | |
| Studies on adjuvant platinum derivate and taxane | | | | | | | | | | | | | | |
| Abe et al.(28) | CRT  CRT+AdjTx | 20  17 | ~83%  90% | ~75%  ~85% | 0.81 | ~60%  60% | ~51%  ~50% | 0.75 | ~85%  ~88% | | .  . | . | HR | |
| Boardman et al.(29) | CRT+AdjTx | 10 | . | . | . | . | . | . | . | | . | . | HR | |
| Kim et al.(30) | CRT+AdjTx | 18 | ~75% | 60% | . | ~66% | 52% | . | . | | ~89% | . | SC | |
| Lorusso et al.(31) | CRT  CRT+AdjTx | 9  10 | .  . | .  . | . | .  . | .  . | . | .  . | | .  . | . | HR | |
| Mabuchi et al.(20) | CRT  CRT+AdjTx | 52  30 | ~71%  ~95% | ~68%  91% | 0.011 | ~57%  ~78% | ~55%  68% | 0.10 | .  . | | .  ~77% | . | HR | |
| Manders et al.(37) | CRT  CRT+AdjTx | 44  7 | ~47%  ~71% | ~31%  ~71% | 0.48 | ~40%  ~53% | .  . | 0.49 | .  . | | .  . | . | HR | |
| Tangjitgamol et al.(33) | CRT  CRT+AdjTx | 129  130 | ~88%  ~80% | 80%  70% | 0.22 | ~70%  ~65% | 67%  63% | 0.29 | ~88%  ~92% | | .  . | . | LR | |
| Tu et al.(34) | CRT  CRT+AdjTx | 38  46 | ~70%  ~88% | ~60%  85% | 0.023 | ~72%  ~84% | ~64%  83% | 0.063 | .  . | | ~63%  ~87% | . | HR | |
| Wang et al.(36) | CRT+AdjTx | 79 | . | ~23% | . | . | . | . | . | | . | . | HR | |
| Yavas et al.(35) | CRT  CRT+AdjTx | 63  46 | 83%  96% | .  . | 0.012 | 83%  94% | .  . | 0.001 | ~87%  ~98% | | .  . | 0.047 | HR | |
| Zhang et al.(38) | CRT+AdjTx | 34 |  | 93% | . | . | . | . | . | | . | . | SC | |

Pandya et al.(32) (adjuvant carboplatin and paclitaxel) did not report results on tumor response or survival outcomes and is therefore not presented in this table. Definition of abbreviations: p = p-value; CRT = chemoradiation, AdjTx = adjuvant systemic therapy; . = not reported or not applicable; NS = not significant; HR = high risk; SC = some concerns; LR = low risk. ^~^ Outcome not reported in text according to Kaplan-Meier method; estimate based on reported crude number of events or deducted from survival graph ^a^ Partial response defined as ≥30% regression instead of >50%.

**Table S9.** Survival outcomes (2/2).

|  |  |  | Overall survival | | | | Recurrence-free survival | | | | Distant metastasis-free survival | | | Risk  of bias |  |
| --- | --- | --- | --- | --- | --- | --- | --- | --- | --- | --- | --- | --- | --- | --- | --- |
| Study | Treatment | N | 2 yr | 3 yr | p | 2 yr | | 3 yr | p | 2 yr | | 3 yr | p |  | |
| Adjuvant cisplatin + ifosfamide | | | | | | | | | | | | | | | |
| Split University | CRT+AdjTx | 118 | ~87% | ~81% | . | ~85% | | ~78% | . | . | | . | . | HR | |
| Adjuvant cisplatin | | | | | | | | | | | | | | | |
| Dubay et al.(39) | CRT+AdjTx | 21 | ~71% | . | . | ~71% | | . | . | . | | . | . | HR | |
| Adjuvant carboplatin | | | | | | | | | | | | | | | |
| Sood et al.(40) | CRT+AdjTx | 25 | . | . | . | . | | . | . | ~95% | | 91% | . | HR | |
| Adjuvant 5-fluorouracil | | | | | | | | | | | | | | | |
| Lorvidhaya et al.(41) | CRT  CRT+AdjTx | 233  230 | ~88%  ~84% | ~84%  ~77% | NS | ~78%  ~76% | | ~68%  ~63% | NS | .  . | | ~84%  ~83% | NS | LR | |
| Adjuvant capecitabin | | | | | | | | | | | | | | | |
| Domingo et al.(42) | CRT+AdjTx | 60 | . | . | . | ~86% | | ~76% | . | . | | . | . | HR | |
| Adjuvant cisplatin + 5-fluorouracil + interferon alpha + retinoic acid | | | | | | | | | | | | | | | |
| Wilailak et al.(48) | CRT+AdjTx | 8 | ~100% | . | . | ~75% | | . | . | ~88% | | . | . | HR | |

The study by Mayadev et al.(49) (adjuvant ipilimumab) only 1-year estimates for progression-free survival (81%) and overall survival (90%) and is therefore not presented in the table. The study by Duska et al.(50) (adjuvant pembrolizumab) does not report survival outcomes and is therefore not presented in the table. Definition of abbreviations: p = p-value; . = not reported or not applicable; NS = not significant; HR = high risk; LR = low risk. ^~^ Outcome not reported in text according to Kaplan-Meier method; estimate based on reported crude number of events or deducted from survival graph.
