# Supplementary material for: Adjuvant Systemic Therapy after Chemoradiation and Brachytherapy for Locally Advanced Cervical Cancer: A Systematic Review and Meta-Analysis
Source: Cancers (Basel). 2021 Apr 14;13(8):1880. doi: 10.3390/cancers13081880 (PMC8070970; doi:10.3390/cancers13081880)
Supplement: Supplementary file 1 [file cancers-13-01880-s001.zip › Cancers-1149973_supplementary/cancers-1149973-supplementary I_XML..docx]

Review

Supplementary Materials II: Adjuvant Systemic Therapy after Chemoradiation and Brachytherapy for Locally Advanced Cervical Cancer: A Systematic Review and Meta-Analysis

Nanda Horeweg ^1,^*, Prachi Mittal ^2^, Patrycja L. Gradowska ^3^, Ingrid Boere ^4^, Supriya Chopra ^5,†^ and Remi A. Nout ^6,†^

Search terms Pubmed, EMBASE and Web of Science

Structure literature searches

“Patients” AND “Primary treatment” AND “Adjuvant treatment” NOT “Exclusion criteria”

1. PubMed

Search string

("Uterine Cervical Neoplasms"[Mesh] OR “cervical neoplasm”[tiab] OR “cervix neoplasm”[tiab] OR “cervical cancer”[tiab] OR “cervix cancer”[tiab] OR “cervical carcinoma”[tiab] OR “cervix carcinoma”[tiab]) AND (“Chemoradiotherapy”[Mesh] OR “Combined Modality Therapy”[Mesh] OR “Radiotherapy”[Mesh] OR “Radiotherapy, Conformal”[Mesh] OR “Radiotherapy, Image-Guided”[Mesh] OR “Radiotherapy, Computer-Assisted”[Mesh] OR “Adenocarcinoma/radiotherapy”[Mesh] OR “Carcinoma, Adenosquamous/radiotherapy”[Mesh] OR “Carcinoma, Squamous Cell/radiotherapy”[Mesh] OR “Chemoradiotherapy”[tiab] OR “Chemoradiation”[tiab] OR “radiotherapy”[tiab] OR “radiation therapy”[tiab] OR “radiochemotherapy”[tiab] **OR** “Brachytherapy”[Mesh] OR “brachytherapy”[tiab]) AND ("Chemotherapy, Adjuvant"[Mesh] OR "Consolidation Chemotherapy"[Mesh] OR "Molecular Targeted Therapy"[Mesh] OR "Immunologic Factors"[Mesh] OR "Lipids/therapeutic use"[Mesh] OR "Mannans/therapeutic use"[Mesh] OR "Antineoplastic Agents, Immunological" [Pharmacological Action] OR "Antibodies, Monoclonal"[Mesh] OR "Antibodies, Neoplasm"[Mesh] OR "Antiviral Agents"[Mesh] OR "Cancer Vaccines"[Mesh] OR "Immunotherapy, Adoptive"[Mesh] OR "Antibodies, Neutralizing"[Mesh] OR "Immunotherapy"[Mesh:NoExp] OR "Carboplatin"[Mesh] OR “Carboplatin”[tiab] OR "Deoxycytidine"[Mesh] OR “Deoxycytidine”[tiab] OR "Taxoids"[Mesh] OR “Paclitaxel”[tiab] OR "gemcitabine" [Supplementary Concept] OR “gemcitabine”[tiab] OR "specific substance maruyama" [Supplementary Concept] OR “Z-100”[tiab] OR “monoclonal antibody”[tiab] OR "Antibodies, Blocking"[Mesh] OR "pembrolizumab" [Supplementary Concept] OR “pembrolizumab”[tiab] OR "Nivolumab"[Mesh] OR “nivolumab”[tiab] OR "cemiplimab" [Supplementary Concept] OR "cemiplimab" [tiab] OR "atezolizumab" [Supplementary Concept] OR "atezolizumab" [tiab] OR "avelumab" [Supplementary Concept] OR "avelumab" [tiab] OR "durvalumab" [Supplementary Concept] OR "durvalumab" [tiab] OR "Ipilimumab"[Mesh] OR "Ipilimumab"[tiab] OR "Trastuzumab"[Mesh] OR "Trastuzumab"[tiab] OR "Cetuximab"[Mesh] OR "Cetuximab"[tiab] OR "Lapatinib"[Mesh] OR "Lapatinib"[tiab] OR "plerixafor octahydrochloride" [Supplementary Concept]) NOT ("Uterine Cervical Neoplasms/surgery"[Mesh] OR "Hysterectomy"[Mesh] OR “hysterectomy”[tiab] OR "Preoperative Period"[Mesh] OR “preoperative”[tiab] OR "Postoperative Period"[Mesh] OR “postoperative”[tiab] OR “MicroRNAs”[Mesh] OR “microRNA”[tiab] OR “Image Interpretation, Computer-Assisted”[Mesh] OR “image interpretation”[tiab] OR “Target delineation”[tiab] OR "Electroacupuncture"[Mesh] OR "Neoadjuvant Therapy"[Mesh] OR “Neoadjuvant”[ALL] OR “neo-adjuvant”[ALL] OR “Induction Chemotherapy”[mesh] OR “induction”[tiab] OR "Agglutinins"[Mesh] OR "Immunosuppressive Agents"[Mesh] OR “morphine”[tiab] OR "Cell Line, Tumor"[Mesh] OR “HIV infections”[mesh] OR “HIV”[ALL] OR “Case reports”[Publication Type] OR “Retracted Publication”[Publication Type] OR “Retraction of Publication”[Publication Type])

Limit on publication date from 2000 onwards.

**Search returned 199 articles on 15-09-2020**

2. EMBASE

Search string:

1. ((exp uterine cervix carcinoma/ or exp uterine cervix cancer/ or "cervical neoplasm".ti,ab. or "cervix neoplasm".ti,ab. or "cervical cancer".ti,ab. or "cervix cancer".ti,ab. or "cervical carcinoma".ti,ab. or "cervix carcinoma".ti,ab.) and (chemoradiotherapy/ or "Chemoradiotherapy".ti,ab. or "Chemoradiation".ti,ab. or "radiochemotherapy".ti,ab. or multimodality cancer therapy/ or cancer radiotherapy/ or image guided radiotherapy/ or intensity modulated radiation therapy/ or external beam radiotherapy/ or volumetric modulated arc therapy/ or "radiotherapy".ti,ab. or "radiation therapy".ti,ab. or brachytherapy/ or "brachytherapy".ti,ab.) and (exp adjuvant chemoradiotherapy/ or consolidation chemotherapy/ or molecularly targeted therapy/ or immunologic factor/ or immunological antineoplastic agent/ or antineoplastic monoclonal antibody/ or antivirus agent/ or cancer vaccine/ or carboplatin/ or deoxycytidine/ or paclitaxel/ or gemcitabine/ or "maruyama".ti,ab. or z 100/ or epidermal growth factor receptor kinase inhibitor/ or "plerixafor octahydrochloride".ti,ab.)) not ("Surgery" or "Hysterectomy" or "preoperative" or "postoperative" or "MicroRNAs" or "image interpretation" or "Target delineation" or "Electroacupuncture" or "Neoadjuvant" or "neo-adjuvant" or "induction" or "Agglutinins" or "morphine" or "Cell Line" or "HIV").mp.
2. limit 1 to (yr=”2000-Current”)

**Search returned 265 articles on 15-09-2020**

3. Web of Science

Search string Web of Science

TS=(Uterine Cervical Neoplasms OR cervical neoplasm OR cervix neoplasm OR cervical cancer OR cervix cancer OR cervical carcinoma OR cervix carcinoma) AND TS=(Chemoradiotherapy OR Chemoradiation OR Radiochemotherapy OR Radiotherapy OR radiation therapy OR Brachytherapy) AND ALL=("adjuvant chemotherapy" OR "Consolidation Chemotherapy" OR "Molecular Targeted Therapy" OR "Immunologic Factors" OR "Mannans" OR "Immunological Antineoplastic Agents" OR "Monoclonal Antibodies" OR "Antiviral Agent" OR "Cancer Vaccine" OR "Immunotherapy" OR "Carboplatin" OR "Deoxycytidine" OR "Taxoids" OR "Paclitaxel" OR "gemcitabine" OR " maruyama" OR "Z-100" OR "monoclonal antibody" OR "Blocking Antibodies" OR "pembrolizumab" OR "Nivolumab" OR "cemiplimab" OR "cemiplimab" OR "atezolizumab" OR "avelumab" OR "durvalumab" OR "Ipilimumab" OR "Trastuzumab" OR "Cetuximab" OR "Lapatinib" OR "plerixafor octahydrochloride") NOT ALL=(surgery OR Hysterectomy OR preoperative OR postoperative OR MicroRNA OR Image Interpretation OR delineation OR contouring OR Electroacupuncture OR Neoadjuvant OR neo-adjuvant OR induction OR Agglutinins OR Immunosuppressive OR morphine OR Cell Line OR HIV OR Case report OR Retracted Publication OR Retraction of Publication)

Limit on publications since 2000

**Search returned 286 articles on 15-09-2020**

4. Total

Systematic search in PubMed returned 199 articles

Systematic search in EMBASE returned 265 articles

Systematic search in Web of Science returned 286 articles

In total 750 articles were found.

138 of 750 appeared to be duplicate articles.

612 unique articles were found.

Data extraction details

Risk of bias

We developed a risk of bias (RoB) tool specifically for this study because there is currently no suitable tool, to our knowledge, for the RoB-assessment of prospective and retrospective studies with or without a control group. Our RoB protocol (Appendix C) is based on the Cochrane Handbook for Systematic Reviews version 5.1.0, and the MOOSE consensus on reporting of observational studies. It describes how to rate risk of selection bias, performance bias, detection bias, attrition bias, reporting bias, registration bias, confounding-by-indication and other ‘biases’ such as claimed fraud or conflicts of interest. An electronic CRF was designed and implemented for the RoB-assessment using Castor EDC. Both review authors (N.H. and P.M.) were trained and a pilot study with a test article conducted. For each included study the two reviewers independently rated all RoB aspects. Studies with discrepancies were listed and discussed, in case of remaining disagreement, the third reviewer was consulted.

Study data

Electronic CRFs were specifically designed and tested in a pilot study with a test-article by S.C. where after some improvements were made. The two review authors (N.H. and P.M. were trained with the final CRF using a different test-article. The two reviewers independently extracted the following data:

- Publication details: author, country, journal, year of publication
- Study design: type of study, methodology, definition of outcomes
- Study population: numbers included, patient and tumor characteristics
- Standard treatment: methods EBRT, brachytherapy and concurrent chemotherapy
- Experimental treatment: methods EBRT, brachytherapy and concurrent chemotherapy, type and schedule of adjuvant systemic therapy
- Summary measures of outcomes: follow-up time, completion rates for EBRT, brachytherapy, concurrent chemotherapy, tumor response, survival outcomes (1, 2, 3 and 5-year actuarial estimates with measures of dispersion, hazard ratio (HR) with 95% confidence intervals (95%CI)) and acute (occurring <3 months) and late (occurring or persisting beyond 3 months) severe toxicity (grade 3-5) outcomes (actuarial estimates with measures of dispersion). If actuarial estimates of survival or toxicity outcomes were not directly reported the estimates were deducted from survival graphs or reported crude numbers.

The data were compared, variables with discrepancies were listed and discussed by the two reviewers and with the third reviewer in case of disagreement.

Risk of bias assessment protocol

This document describes the risk of bias assessment for the systematic review on adjuvant systemic therapy in locally advanced cervical cancer. A tailored risk of bias assessment tool was developed for this review because a great variety of studies was included; both randomized and non-randomized studies wherein standard concurrent chemoradiation (CRT) is being compared to CRT followed by adjuvant systemic therapy (Adj Tx) and (retro and prospective) studies that only report on a group treated with CRT + Adj Tx.

This tool only addresses to forms of bias that could affect the intrinsic validity of a study (reliability of the study estimates given its study population and design). Biases that could affect the extrinsic validity of a study (affecting for example extrapolation of the results to the general patient population of interest) are not part of this risk of bias assessment, but will be addressed to in the discussion section of the article; as recommended by the Cochrane Handbook.

The risk of bias assessment for the included randomized controlled trials will be performed according to the Cochrane handbook of systematic reviews (version 5.1.0). The risk of bias assessment of the included non-randomized interventional and observational studies will be performed in accordance with the MOOSE consensus on reporting of observational studies (Stroup et al. for the 'Meta-analysis of observational studies in Epidemiology (MOOSE) group, JAMA 2000).

The following forms of bias are deemed important for the quality assessment of the studies included in the systematic review:

| **Type of bias** | **Description** | **Relevant domains for Risk of Bias assessment** | | |
| --- | --- | --- | --- | --- |
|  |  | **Randomized controlled trials** | **Prospective non-randomized studies** | **Retrospective studies** |
| Selection bias | Systematic differences between baseline characteristics of the groups that are compared. | - Sequence generation  - Allocation concealment  - Per protocol analysis instead of intention to treat analysis  - Exclusion of patients for events occurring after start treatment | - Per protocol analysis instead of intention to treat analysis  - Exclusion of patients for events occurring after start treatment | - Per protocol analysis instead of intention to treat analysis  - Exclusion of (not including) patients for events occurring after start treatment or availability or completeness of follow-up data |
| Performance bias | Systematic differences between groups in the care that is provided, or in exposure to factors other than the interventions of interest. | - Blinding of participants and personnel.  - Differences in treatment or care setting between groups other than the invention of interest  - Other potential threats to validity. | - Differences in treatment or care setting between groups other than the invention of interest | - Differences in treatment or care setting between groups other than the invention of interest |
| Detection bias | Systematic differences between groups in how outcomes are determined. | - Blinding of outcome assessment.  - Differences between groups in follow-up strategy and endpoint assessment and registration  - Other potential threats to validity. | - Blinding of outcome assessment.  - Differences between groups in follow-up strategy and endpoint assessment and registration | - Blinding of outcome assessment.  - Differences between groups in follow-up strategy and endpoint assessment and registration |
| Attrition bias | Systematic differences between groups in withdrawals from a study. | Incomplete outcome data | Incomplete outcome data | Incomplete outcome data |
| Reporting bias | Systematic differences between reported and unreported findings. | Outcomes for endpoints not prespecified or selectively reported | Outcomes for endpoints not prespecified or selectively reported | Bias inherent to design |
| Registration bias | Incomplete registration of study data (including recall bias) may causing residual confounding | In case data registration was not entirely predefined and prospective | In case data registration was not entirely predefined and prospective | Bias inherent to design |
| Confounding by indication bias | Factors that are related to study outcomes have influenced treatment allocation | Should not be the case if treatment was correctly randomly allocated | In case patients or doctors chose between treatment options | Bias inherent to design |
| Other biases |  | - Fraud, conflicts of interest | - Fraud, conflicts of interest | - Fraud, conflicts of interest |

Below, the criteria for judging risk of each of the aforementioned biases are provided. Using the descriptions, one should determine whether the risk of bias of an included study is low, high or unclear.

Selection bias

Should be judged according to one or more of the following domains, depending on the type of study.

- **Random treatment allocation (only for randomizes studies)**

Lack of an adequate method to randomly allocate treatment to patients can introduce selection bias. Classify the risk of bias using the following criteria:

Low risk: The investigators describe a random component in the sequence generation process such as: referring to a random number table; using a computer random number generator; coin tossing; shuffling cards or envelopes; throwing dice; drawing of lots; minimization (may be implemented without a random element, and this is considered to be equivalent to being random).

High risk: The investigators describe a non-random component in the sequence generation process. Usually, the description would involve some systematic, non-random approach, for example: sequence generated by odd or even date of birth; sequence generated by some rule based on date (or day) of admission; or sequence generated by some rule based on hospital or clinic record number.

Other non-random approaches usually involve judgement or some method of non-random categorization of participants, for example: allocation by judgement of the clinician; allocation by preference of the participant; allocation based on the results of a laboratory test or a series of tests; allocation by availability of the intervention.

In addition, all studies that were designed as a non-randomized comparative study should be considered as at high risk for selection bias. Examples are comparative prospective cohorts, comparative retrospective cohort studies, comparison of historical cohorts (e.g. cohort before compare to cohort after the implementation of a new treatment feature).

Unclear risk: Insufficient information about the treatment allocation process to permit judgement of ‘Low risk’ or ‘High risk’.

- **Allocation concealment (only for randomizes studies)**

If the allocation of the treatment was not adequately concealed (hidden) prior to the inclusion of the patient in the study, selection bias could have been introduced. Classify the risk of bias using the following criteria:

Low risk: Both the patients and the investigator who enrolled the patient could not foresee assignment because one of the following, or an equivalent method, was used to conceal allocation: central allocation (including telephone, web-based and pharmacy-controlled randomization); sequentially numbered drug containers of identical appearance; sequentially numbered, opaque, sealed envelopes.

High risk: The patients and/or the investigator who enrolled the patient could possibly foresee assignments and thus introduce selection bias, such as allocation based on: using an open random allocation schedule (e.g. a list of random numbers); assignment envelopes were used without appropriate safeguards (e.g. if envelopes were unsealed or non-opaque or not sequentially numbered); alternation or rotation; date of birth; case record number; any other explicitly unconcealed procedure.

In addition, all studies that were designed as a non-randomized comparative study should be considered as at high risk for selection bias. Examples are comparative prospective cohorts, comparative retrospective cohort studies, comparison of historical cohorts (e.g. cohort before compare to cohort after the implementation of a new treatment feature).

Unclear risk: Insufficient information to permit judgement of ‘Low risk’ or ‘High risk’. This is usually the case if the method of concealment is not described or not described in sufficient detail to allow a definite judgement – for example if the use of assignment envelopes is described, but it remains unclear whether envelopes were sequentially numbered, opaque and sealed.

- **Type of outcome analysis: intention-to-treat vs. per-protocol analysis**

If the outcomes of the study were analyzed by per-protocol analysis instead of by intention-to-treat analysis, selection bias could have been introduced.

Description intention-to-treat analysis: the treatment groups that are being compared in this type of analysis, consist of all patients who were supposed to get the treatment assigned at inclusion/randomization; this includes patients who, at a later stage, crossed over to the other study arm and/or patients who did not receive treatment.

Description per-protocol analysis: also called 'as treated analysis'. The treatment groups that are being compared in this type of analysis, consist of the groups of patients who actually received the treatment; independent of the treatment they were supposed to get at inclusion/randomization.

Classify the risk of bias using the following criteria:

Low risk: Any of the following: 1) intention-to-treat analysis were performed; or 2) per-protocol analysis were performed together with proper sensitivity analysis that showed that the outcomes of the per-protocol analysis are not different from the outcomes of an intention-to-treat analysis.

High risk: Per-protocol analysis were performed, without a proper sensitivity analysis that showed that the outcomes of the per-protocol analysis are not different from the outcomes of an intention-to-treat analysis.

Unclear risk: Insufficient information to permit judgement of ‘Low risk’ or ‘High risk’. This is usually the case if the method of analysis is not described or not described in enough detail to allow a definite judgement.

- **Exclusion of patients for events occurring after start of treatment**

Selection bias could have been introduced if patients were excluded from statistical analysis because of events occurring after start of treatment (such as not completing treatment, toxicity, comorbid events) or the availability/completeness of follow-up or outcome data.

This type of bias is very often present in retrospective studies, as patients are often included based criteria like having completed the treatment of interest or having available follow-up data.

Classify the risk of bias using the following criteria:

Low risk: No exclusion of patients for analysis because of events occurring after the start of treatment or because of missing follow-up or outcome data.

High risk: Exclusion of patients for analysis because of events occurring after the start of treatment or because of missing follow-up or outcome data.

Unclear risk: Insufficient information to permit judgement of ‘Low risk’ or ‘High risk’.

Performance bias

Should be judged according to one or more of the following domains, depending on the type of study.

- **Blinding of participants and personnel (only for prospective studies)**

Performance bias may be introduced when knowledge on the allocated treatment by the patient and/or personnel influences the outcome of the study. Classify the risk of bias using the following criteria: differences between groups in the care that is provided, or in exposure to factors other than the interventions of interest.

Low risk: Any of the following: 1) no blinding or incomplete blinding, but the review authors judge that the care for the patient and exposure to factors other than the allocated treatment is not likely to be influenced by lack of blinding; or 2) Blinding of participants and key study personnel ensured, and unlikely that the blinding could have been broken.

High risk: Any of the following: 1) no blinding or incomplete blinding, and the review authors judge that the care for the patient and/or exposure to factors other than the allocated treatment is likely to be influenced by lack of blinding; or 2) blinding of key study participants and personnel attempted, but likely that the blinding could have been broken, and that the care for the patient and/or exposure to factors other than the allocated treatment outcome are likely to be influenced by lack of blinding; or 3) the care for the patient and/or exposure to factors other than the allocated treatment were different in the treatment groups (e.g. different care settings or different periods of time)

Unclear risk: insufficient information to permit judgement of ‘Low risk’ or ‘High risk.

- **Differences in treatment between groups other than the invention of interest**

Performance bias may be introduced by differences in treatment or care setting between groups other than the invention of interest. Examples are the groups are not treated in the same hospitals, the groups are not treated in the same time periods (e.g. comparison to a historical cohort), the groups did not receive the same supportive care alongside or after treatment etc.

Classify the risk of bias using the following criteria:

Low risk: 1) the treatment and/or the setting wherein the patient was treated and/or exposure to factors other than the allocated treatment is not likely to be different between study groups or 2) the care for the patient and exposure to factors other than the allocated treatment is different between study groups, but the review authors judge that it is unlikely that this has introduced bias.

High risk: the treatment and/or the setting wherein the patient was treated and/or exposure to factors other than the allocated treatment is likely to be different between (in within for single arms studies) study groups, and the review authors judge it is likely to have introduced bias.

Unclear risk: 1) insufficient information to permit judgement of ‘Low risk’ or ‘High risk’; or 2) the study did not address this outcome.

Detection bias

Should be judged according to one or more of the following domains:

- **Blinding of outcome assessment**

Detection bias may be introduced when the allocated interventions is known by outcome assessors.

Classify the risk of bias using the following criteria:

Low risk: Any of the following: 1) blinding of outcome assessment ensured, and unlikely that the blinding could have been broken; or 2) no blinding of outcome assessment, but the review authors judge that the outcome measurement is not likely to be influenced by lack of blinding.

High risk: Any of the following: 1) no blinding of outcome assessment, and the outcome measurement is likely to be influenced by lack of blinding; or 2) blinding of outcome assessment, but likely that the blinding could have been broken, and the outcome measurement is likely to be influenced by lack of blinding.

Unclear risk: 1) Insufficient information to permit judgement of ‘Low risk’ or ‘High risk’.

- **Differences between groups in follow-up strategy and endpoint assessment**

Detection bias may be introduced when the follow-up strategy and/or endpoint determination and/or registration were not the same in the treatment groups.

Examples are: the frequency of follow-up visits is not the same in the treatment groups, or the diagnostic procedure to assess treatment failure is not the same in the treatment groups (e.g. CT vs. MRI), or the data source of the study endpoints is not the same in the treatment groups (e.g. electronic patient files vs. national registries).

Classify the risk of bias using the following criteria:

Low risk: Any of the following: 1) no differences in follow-up strategy and/or endpoint determination and/or registration between treatment groups; or 2) difference(s) in follow-up strategy and/or endpoint determination and/or registration blinding of outcome between groups, but the review authors judge that this is not likely to have influenced outcome assessment.

High risk: differences in follow-up strategy and/or endpoint determination and/or registration between treatment groups, and the outcome measurement is likely to be influenced by lack of blinding.

Unclear risk: Insufficient information to permit judgement of ‘Low risk’ or ‘High risk’.

Attrition bias

Should be judged according to the following domain:

- **Incomplete outcome data**

Attrition bias may be introduced when there are differences between the groups in the completeness of outcome data. Reasons for this may be informative censoring (e.g. more lost-to-follow-up in the group with a more intense treatment).

Classify the risk of bias using the following criteria:

Low risk: Any of the following: 1) No missing outcome data; or 2) missing outcome, but review authors judge that reasons for missing outcome data are unlikely to be related to true outcome (for survival data, censoring unlikely to be introducing bias); or 3) Missing outcome data balanced in numbers across intervention groups, with similar reasons for missing data across groups; or 4) For dichotomous outcome data, the proportion of missing outcomes compared with observed event risk not enough to have a clinically relevant impact on the intervention effect estimate; 5) For continuous outcome data, plausible effect size (difference in means or standardized difference in means) among missing outcomes not enough to have a clinically relevant impact on observed effect size; or 6) Missing data have been imputed using appropriate methods.

High risk: Any of the following: 1) Outcome data is incomplete and reasons for missing outcome data are likely to be related to true outcome, with either imbalance in numbers or reasons for missing data across intervention groups; or 2) For dichotomous outcome data, the proportion of missing outcomes compared with observed event risk enough to induce clinically relevant bias in intervention effect estimate; 3) For continuous outcome data, plausible effect size (difference in means or standardized difference in means) among missing outcomes enough to induce clinically relevant bias in observed effect size; or 4) ‘As-treated’ analysis done with substantial departure of the intervention received from that assigned at randomization; or 5) Potentially inappropriate application of simple imputation.

Unclear risk: Insufficient reporting of attrition/exclusions to permit judgement of ‘Low risk’ or ‘High risk’ (e.g. number randomized not stated, no reasons for missing data provided).

Reporting bias

Should be judged according to the following domain:

- **Selective outcome reporting**

Reporting bias may be introduced when there are systematic differences between reported and unreported findings between groups.

Classify the risk of bias using the following criteria:

Low risk: Any of the following: 1) The study protocol is available and all of the study’s pre-specified (primary and secondary) outcomes that are of interest in the review have been reported in the pre-specified way; or 2) The study protocol is not available but it is clear that the published reports include all expected outcomes, including those that were pre-specified (convincing text of this nature may be uncommon).

High risk: Any of the following: 1) Not all of the study’s pre-specified primary outcomes have been reported; or 2) One or more primary outcomes is reported using measurements, analysis methods or subsets of the data (e.g. subscales) that were not pre-specified; or 3) One or more reported primary outcomes were not pre-specified (unless clear justification for their reporting is provided, such as an unexpected adverse effect); or 4) One or more outcomes of interest in the review are reported incompletely so that they cannot be entered in a meta-analysis; or 5) The study report fails to include results for a key outcome that would be expected to have been reported for such a study.

Unclear risk: Insufficient information to permit judgement of ‘Low risk’ or ‘High risk’.

Registration bias

Should be judged according to the following domain:

- **Incomplete or retrospective data collection**

Registration bias may be introduced when study data was incompletely or retrospectively registered and/or collected (including recall bias). This may cause residual confounding. Registration bias is inherent to retrospective studies.

Classify the risk of bias using the following criteria:

Low risk: Any of the following: 1) All study data were pre-defined and prospectively collected; or 2) Not all study data were pre-defined and prospectively collected, but complete data collection was secured in a retrospective fashion and the review authors judge that this is not likely to have causes registration bias.

High risk: Any of the following: 1) Not all study data were pre-defined and prospectively collected and the review authors judge that this has likely to have caused registration bias.

Unclear risk: Insufficient information to permit judgement of ‘Low risk’ or ‘High risk’.

Confounding by indication bias

Should be judged according to the following domain:

Confounding by indication bias may be introduced when patients were assigned to a treatment based on factors that are related to the study outcomes. This type of bias should not be present in randomized studies, if treatment was correctly randomly allocated. This type of bias could be present in non-randomized prospective studies and is inherent to retrospective studies.

Classify the risk of bias using the following criteria

Low risk: Any of the following: 1) Treatment was correctly randomly assigned; or 2) Treatment was not randomly assigned but the review authors judge that it is not likely that factors related to the study outcomes have played a role in treatment assignment.

High risk: Any of the following: 1) Treatment was not randomly assigned and the review authors judge that it is likely that factors related to the study outcomes have played a role in treatment assignment; or 2) Retrospective inclusion of patients based on received treatment.

Unclear risk: Insufficient information to permit judgement of ‘Low risk’ or ‘High risk’.

Other bias

Bias due to fraud, conflicts of interest or other problems not covered elsewhere.

Low risk: There are no indications that study has been subject to fraud or that any of the authors have conflicts of interest that could have influenced the results presented, neither are there other problems that are not covered elsewhere in this risk of bias assessment.

Some concerns: It cannot be excluded that suspected fraud, conflicts of interest or other problems not addressed to elsewhere in the risk of bias assessment have biased the results of the study.

High risk: The study has been confirmed to be fraudulent or conflicts of interest have biased the study’s outcomes; or some other problem has caused bias.

Unclear risk: There may be a risk of bias, but there is either: insufficient information to assess whether an important risk of bias exists; or insufficient rationale or evidence that an identified problem will introduce bias.

Overall risk of bias

Low risk: The study is judged to be at low risk of bias in all domains.

Some concerns: The study is judged to raise some concerns of bias because there are unclarities, but no domains have been classified as at high risk of bias.

High risk: The study is judged to be at high risk of bias due to a high risk of bias in one or more domains.

Supplementary statistical methods

Imputations

Calculation of a pooled hazard ratio (HR) requires knowledge of HRs and their corresponding variances for all studies included in the meta-analysis. If the included articles did not provide this, these were imputed using other available data using the methodology proposed by Tierney (Tierney et al. Trials. 2007). In their article, 11 different strategies to estimate HR and its variance (V) based on published data are described, of which four were applied in the current study. The calculated HRs and their variance were used to derive lnHR and its variance (if applicable), which are the actual input to the meta-analysis, as follows:

**Eq.1**

| $\ln HR=ln(HR)$ | (1) |
| --- | --- |

**Eq.2**

| $Variance of the\ln HR= \frac{1}{Variance of the HR (V)}$ | (2) |
| --- | --- |

The different formulas used for calculating HR and/or its variance are provided below.

Report presents HR and its confidence interval (CI) (Strategy 3)

**Eq.3**

| $Variance of the\ln HR= \left( \frac{\ln\left( upper CI \right)-\ln(lower CI)}{2\times z score for upper CI boundry} \right)^{2}$ | (3) |
| --- | --- |

Report presents p-value and event in each arm and the randomization ratio is 1:1 (Strategy 7)

**Eq.4**

| $O-E= \sqrt{\frac{Observed events CRT\times Observed events AdjTx}{Total observed events}} \times(z score for p value \div2)$ | (4) |
| --- | --- |

A positive or negative value for O – E (Observed – Expected) is assigned based on the direction of the effect reported in the study.

**Eq.5**

| $Variance \left( V \right)= \frac{Observed events AdjTx\times Observed events CRT}{Total events}$ | (5) |
| --- | --- |

**Eq.6**

| $HR=\exp\left( \frac{O-E}{V} \right)$ | (6) |
| --- | --- |

Report presents p-value, total events and numbers randomized to each arm and the randomization ratio is not 1:1 (Strategy 9)

**Eq.7**

| $O-E=\sqrt{\frac{(Total observed events\times Analysed AdjTx \times Analysed CRT)}{(Analysed AdjTx+Analysed CRT)}} \times(z score for p value \div2)$ | (7) |
| --- | --- |

A positive or negative value for O – E (Observed – Expected) is assigned based on the direction of the effect reported in the study.

**Eq.8**

| \| $Variance \left( V \right)= \frac{Total observed events \times Analysed AdjTx\times Analysed CRT}{{(Analysed AdjTx+analysed CRT)}^{2}}$ \| (8) \| \| --- \| --- \| | (8) |
| --- | --- | --- | --- |

**Eq.9**

| $HR=\exp\left( \frac{O-E}{V} \right)$ | (9) |
| --- | --- |

Report presents Kaplan-Meier curve and information on follow-up (Strategy 10)

Here, the follow-up time was divided in multiple intervals wherein at least 1, but not more than 20% of the events have occurred. For each interval the numbers event free at the start of the interval (1), numbers censored during the interval (2), numbers at risk during the interval (3) and numbers of events during the interval (4) were calculated using data on follow-up time and percentages deducted from the survival graphs. With all these numbers a HR, variance and O-E were calculated for each interval (5), thereafter these were combined (6).

Step 1: Numbers event-free at the start of the current interval

**Eq.10**

| \| $Event free at start of current interval=Event free at start of prior interval-Events in prior interval-Censored during prior interval$ \|  \| \| --- \| --- \| | (10) |
| --- | --- | --- | --- |

Step 2: Numbers censored during the current interval

**Eq.11**

| $Censored during current interval= At risk during the current interval\times\left( \frac{1}{2}\times\frac{(End of interval-start of interval)}{(maximum follow up-start of time interval)} \right)$ | (11) |
| --- | --- |

Step 3. Numbers at risk during the current interval, adjusted for censoring

**Eq.12**

| $At risk during current interval, adjusted for censoring=Event free at start of current interval-Censored during current interval$ | (12) |
| --- | --- |

Step 4. Number of events during the current interval

**Eq.13**

| $Events in current interval=At risk during current interval\times\left( \frac{\% event free at start-\% event free at end}{\% event free at start} \right)$ | (13) |
| --- | --- |

Step 5. Estimate the HR, V and O-E for the current interval

**Eq.14**

| $HR= \left( \frac{{Events AdjTx}/{At risk AdjTx}}{{Events Events CRT}/{At risk CRT}} \right)$ | (14) |
| --- | --- |

**Eq.15**

| \| $V= \frac{1}{\left( 1/{events AdjTx} \right)-\left( 1/{at risk AdjTx} \right)+\left( 1/{events CRT} \right)-\left( 1/{at risk CRT} \right)}$ \| ) \| \| --- \| --- \| | (15) |
| --- | --- | --- | --- |

**Eq.16**

| $O-E=\ln HR\times V$ | (16) |
| --- | --- |

Step 6. combining all time intervals

The pooled O – E is equal to the sum of the O – E’s of all intervals. The pooled variance (V) is equal to the sum of the variances of all intervals whereas HR is estimated as

**Eq.17**

| $HR=\exp\left[ \frac{\Sigma O-E}{\Sigma V} \right]$ | (17) |
| --- | --- |

The table below shows which of the aforementioned strategies was used for each article.

**Imputation strategy and results by study.**

|  |  |  | **OS** | | | **RFS** | | | **DMFS** | | | |
| --- | --- | --- | --- | --- | --- | --- | --- | --- | --- | --- | --- | --- |
| **Study** | **Year** | **AdjTx** | **Method** | **HR** | **V** | **Method** | **HR** | **V** | | **Method** | **HR** | **V** |
| Duenas-Gonzáles et al.(11) | 2011 | PtPA | 3 | 0.68^a^ | 35.06 | 3 | 0.68^a^ | 35.06 | | 7 | 0.35 | 13.89 |
| Kim et al.(18) | 2008 | PtPA | 10 | 0.92 | 12.61 | 10 | 0.98 | 13.17 | | none |  |  |
| Choi et al.(17) | 2011 | PtPA | 10 | 0.27 | 3.10 | 7 | 0.54 | 6.15 | | 7 | 0.31 | 2.25 |
| Fabri et al.(27) | 2019 | PtPA | 3 | 0.36^a^ | 5.11 | 3 | 0.40^a^ | 9.10 | | 3 | 0.40^a^ | 7.19 |
| Kim et al.(23) | 2007 | PtPA | 10 | 1.38 | 8.38 | 7 | 0.91 | 8.97 | | 7 | 1.00 | 3.73 |
| Kong et al.(19) | 2012 | PtPA | 10 | 1.27 | 12.11 | 9 | 1.02 | 14.45 | | none |  |  |
| Tangjitgamol et al.(33) | 2019 | PtTax | 3 | 1.42^a^ | 12.18 | 3 | 1.26^a^ | 20.24 | | none |  |  |
| Abe et al.(28) | 2012 | PtTax | 10 | 0.71 | 1.00 | 7 | 0.97 | 3.077 | | none |  |  |
| Mabuchi et al.(20) | 2017 | PtTax | 10 | 0.20 | 1.92 | 10 | 0.59 | 6.32 | | none |  |  |
| Manders et al.(37) | 2018 | PtTax | 10 | 0.62 | 1.43 | 10 | 0.76 | 2.75 | | none |  |  |
| Tu et al.(34) | 2017 | PtTax | 9 | 0.35 | 6.19 | 9 | 0.49 | 5.95 | | none |  |  |
| Yavas et al.(35) | 2019 | PtTax | 9 | 0.06 | 1.22 | 10 | 0.19 | 1.83 | | 9 | 0.26 | 2.20 |

Definition of abbreviations: AdjTx = adjuvant therapy; OS = overall survival; HR = hazard ratio; V = variance; RFS = recurrence-free survival; DMFS = distant metastasis-free survival; PtPA = platinum derivate - pyrimidine antagonist doublet therapy; PtTax = platinum derivate - taxane doublet therapy. ^a^ Value reported in the article.

Details on statistical software

Analyses were performed in Microsoft Excel and R version 3.6.1 (<http://www.r-project.org/>).

R packages used in this study included:

ggplot2: <https://cran.r-project.org/web/packages/ggplot2/index.html>

rjags: <https://cran.r-project.org/web/packages/rjags/index.html>

coda: <https://cran.r-project.org/web/packages/coda/index.html>

mcmcplots: <https://cran.r-project.org/web/packages/mcmcplots/index.html>

metafor: <https://cran.r-project.org/web/packages/metafor/index.html>

metaviz: <https://cran.r-project.org/web/packages/metaviz/index.html>
